# Exploring the Cytokinin Profile of *Doliocarpus dentatus* (Aubl.) Standl. From Guyana and Its Relationship with Secondary Metabolites: Insights into Potential Therapeutic Benefits

**DOI:** 10.3390/metabo15080533

**Published:** 2025-08-06

**Authors:** Ewart A. Smith, Ainsely Lewis, Erin N. Morrison, Kimberly Molina-Bean, Suresh S. Narine, R. J. Neil Emery

**Affiliations:** 1 Environmental and Life Sciences Graduate Program, Trent University, Peterborough, ON K9J 0G2, Canada; erinnmorrison@trentu.ca (E.N.M.); kimberlymolinabean@trentu.ca (K.M.-B.); 2 Department of Biology, Trent University, Peterborough, ON K9J 0G2, Canada; nemery@trentu.ca; 3 Department of Chemical and Physical Sciences, University of Toronto Mississauga, Mississauga, ON L5L 1C6, Canada; 4 Trent Centre for Biomaterials Research, Trent University, Peterborough, ON K9J 0G2, Canada; sureshnarine@trentu.ca; 5 Departments of Physics & Astronomy and Chemistry, Trent University, Peterborough, ON K9J 0G2, Canada

**Keywords:** Capadulla, cytokinins, *Doliocarpus dentatus*, ethnobotany, Guyana, hormonomics, mass spectrometry, metabolite, metabolomics, orbitrap, polyphenols, traditional therapy

## Abstract

**Background/Objectives**: Possessing red and white ecotypes, and utilized in traditional Guyanese medicine, *Doliocarpus dentatus*’ red ecotype is preferred locally for its purported superior therapeutic efficacy. Although therapeutic metabolites were detected in *D. dentatus* previously, phytohormones remain largely unexplored, until now. Cytokinins, phytohormones responsible for plant cell division, growth and differentiation, are gaining traction for their therapeutic potential in human health. This study screened and quantified endogenous cytokinins and correlated detected cytokinins with selected secondary metabolites. **Methods:** Liquid chromatography–mass spectrometry was used to acquire phytohormone and metabolite data. Bioinformatics tools were used to assess untargeted metabolomics datasets via statistical and pathway analyses, and chemical groupings of putative metabolites. **Results**: In total, 20 of the 35 phytohormones were detected and quantified in both ecotypes, with the red ecotype displaying higher free base and glucoside cytokinin concentrations and exhibited 6.2 times the total CK content when compared to the white ecotype. Pathway analysis revealed flavonoid and monoterpenoid biosynthesis in red and white ecotypes, respectively. Positive correlations between specific cytokinins and alkaloids, and between *trans*-Zeatin and isopentenyladenosine riboside with phenolic compounds were observed. **Conclusions:** These results suggest that the red ecotype’s elevated cytokinin levels coupled with flavonoid biosynthesis enrichment support its preference in Guyanese traditional medicine.

## 1. Introduction

*Doliocarpus dentatus* (Aubl.) Standl. [1], otherwise known as Capadulla, Kapadula, Kabuduli, and as a Guyanese natural aphrodisiac, is a woody vine/liana within the Dilleniaceae family having a significant role in Guyanese traditional medicinal practices [2,3]. Possessing the highest population density in the Amazon, its native habitat spans from Mexico to Southern Brazil and covers areas in Central to South America [4]. *D. dentatus*’ vine flourishes in both mixed and dry Guyanese evergreen forests [5,6,7].

**Figure 1:**
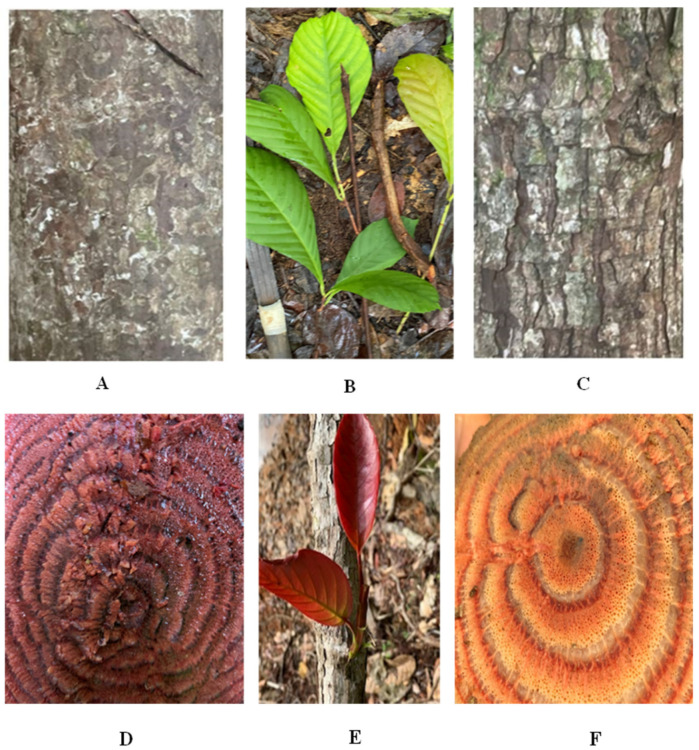
Morphological features of *D. dentatus*’ red and white ecotypes morphological features. (**A**). *D. dentatus* red outer bark, (**B**) mature leaves of both ecotypes, (**C**) *D. dentatus*’ white outer bark, (**D**) *D. dentatus* red inner bark, (**E**) *D. dentatus*’ young leaves of both ecotypes and (**F**) *D. dentatus*’ white inner bark. Photos taken by Ewart Smith (21N0261909-0578704—Potaro Siparuni Region, 8 August 2021, Guyana).

*D. dentatus* has red and white ecotypes (Figure 1), distinguished by their morphological and physical characteristics, such as the texture of the vine’s outer bark and the colour of the xylem and phloem visible during harvesting [8,9]. *D. dentatus*’ red ecotype is preferred in Guyanese traditional medicine to treat various illnesses including malaria, cystitis, erectile dysfunction, cancer and leishmanial ulcers, and used as a contraceptive and disinfectant [8,10,11,12,13]. Popularly utilized by Guyanese men for its purported aphrodisiac properties, *D. dentatus* red ecotype is believed to enhance libido and treat male impotence when consumed as tea or cold drink [9,14,15].

Other Dilleniaceae genera are researched extensively due to their unique ethnobotanical uses and therapeutic potential, characterized by a diverse array of secondary metabolites, including flavonoids, terpenoids, lignans, and phenolic derivatives [3,13,16,17,18,19,20,21,22,23]. Dilleniaceae members were reported to exhibit anti-inflammatory, antioxidant, antimicrobial, and anti-ulcer properties, which can be primarily attributed to the presence of flavonoids and terpenoids [3,9,13,24,25].

Among plant metabolite diversity, phytohormones are plant derived organic signalling molecules that regulate all plant processes including growth, development, source/sink transitions, and nutrient distribution; moreover, they function as key mediators in plant responses to various biotic and abiotic stresses and enable plants to adapt to their dynamic environment [26,27]. These regulatory metabolites are synthesized in both root and aerial plant organs in small concentrations (<10^−8^ M) [26,28,29,30]. Extensive research is ongoing for different phytohormone classes (i.e., abscisic acid, auxins, brassinosteroids, cytokinins, ethylene, gibberellins, jasmonates, and strigolactones; [27,31,32,33,34,35,36,37,38,39]), well known for their essential roles in controlling plant growth, development, and reactions to environmental stimuli across disciplines such as biotechnology, agriculture, and horticulture [40,41].

**Figure 2:**
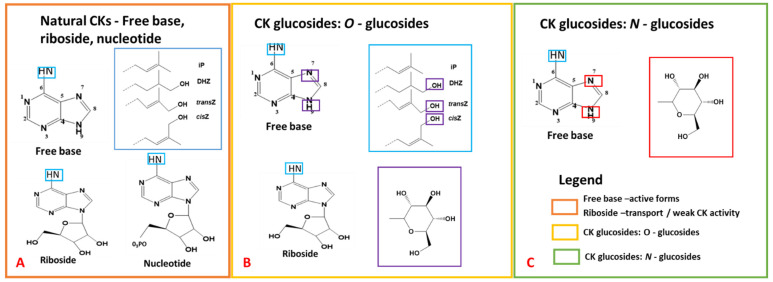
Chemical structures of cytokinins (CKs), adapted and modified from previously published work [50,87]. (**A**) exhibits the adenine structure, and the numbering system utilized for CK nomenclature, encompassing free base, riboside, and nucleotide CKs. (**B**) outlines CK O-glucosides, and (**C**) demonstrates N-glucosides. Blue boxes highlight the incorporation of side chains, with larger blue boxes indicating the various side chains. For O-glucosides, blue boxes indicate the glucose structure and glycosylation sites. Similarly, purple and red boxes indicate the glucose structure and glycosylation sites for N-glucosides.

Recently, increasing emphasis is placed on investigating cytokinins (CKs), their related conjugates, and how they interact with key plant secondary metabolites such as polyphenols, flavonoids, terpenoids, and alkaloids [42,43,44,45,46,47]. CKs are adenine derivatives with an isoprenoid or aromatic side chain attached to the N^6^ position (Figure 2; [48,49,50]. CKs exhibit a variety of physiological functions, with subtle structural changes on compound moiety allowing for fine tune regulation of various plant physiological processes [51], especially in cell division, differentiation, photosynthesis, and nutrient distribution to actively growing sites [40,48,52].

Naturally occurring isoprenoid type CKs include: isopentenyladenine (iP), isomers *trans*-Zeatin (*t*Z) and *cis*-Zeatin (cZ), and dihydrozeatin (DZ) and their riboside forms (Figure 2: [53,54,55]). Although less prevalent naturally than isoprenoid type CKs, aromatic CKs (for example: ortho-topolin, benzyladenine, and kinetin: [53,54,55]) are often used in bioassays due to increased biological activity [50]. Further modifying CK structures (i.e., via glycosylation, xylosylation, amino acid attachment, thiolation, etc.) results in different CK forms (i.e., riboside, nucleotide, and methylthiolated derivatives as well as downstream sugar conjugates; [35,53,54,56]), which help to regulate their levels and activity [57,58,59,60,61,62,63].

Emerging evidence suggests that intricate relationships between CKs, and secondary metabolites exist through complex signaling pathways and biochemical processes in plants. Understanding these interactions can aid in developing new strategies to enhance crop yield, improve plant stress tolerance, understand therapeutic benefits and manage plant growth in various environmental conditions [64]. CKs interacting with secondary metabolites is a growing area of interest in plant biology, as these interactions can influence secondary metabolite biosynthesis and ultimately the overall plant metabolic profile [65]. CKs were shown to influence secondary metabolite biosynthetic pathways, including flavonoids, phenolic compounds, and alkaloids [40,65,66]. Previous studies demonstrated that CKs have significant effects on many genes involved in secondary metabolism, especially in flavonoid biosynthesis which are important for plant defence and pigmentation [67,68,69].

Despite the rapid progress and usage of mass spectrometry-based metabolomics to screen valuable plant compounds [70,71,72], a significant lack of understanding exists regarding establishing *D. dentatus*’ phytohormone profile and its association with secondary metabolites. The metabolic network involving CKs and secondary metabolites can be complex, as CKs not only influence secondary metabolite biosynthesis, but are also subject to multidirectional regulatory relationships including degradation, transport, and perception; all influenced by environmental signals and the internal stages of plant development [73,74,75]. This dual dynamic—where CKs not only regulate secondary metabolite production but are also subject to regulation by various physiological and environmental factors—fosters a highly adaptive and responsive network [29,76,77,78].

Although phytohormones and other secondary metabolites have synergy, phytohormones solely present potential therapeutic benefits for humans [55,79]. Phytohormones (i.e., salicylic acid, abscisic acid, and jasmonic acid) exhibit antioxidant properties that protect cells from free radical damage [80,81]. In human and animal studies, CKs and derivatives demonstrate a range of effects at both the cellular and organismal levels, indicating potential therapeutic applications, including efficacy in cancer treatment [82]. For example, CK ribosides demonstrated anticancer activity in both in vitro and in vivo studies [83]. Additionally, CKs may exhibit antioxidant properties, thereby protecting against oxidative damage and enhancing cellular viability [84,85]. For example, the aromatic CK kinetin reduced apoptosis by protecting cells against oxidative stress at low doses (100 nM; [86]). With various studies reporting CK’s therapeutic effects, it is imperative to screen for and quantify endogenous phytohormone levels in *D. dentatus* to establish a phytohormone profile.

Given the importance of understanding the complex interactions of phytohormones with other secondary metabolites, our research aims to expand this knowledge focusing specifically on *D. dentatus* ecotypes. The study’s primary objective was to screen and quantify endogenous CK levels of *D. dentatus* ecotypes and to investigate their correlation with various secondary metabolites, including alkaloids, flavonoids, and other phenolic compounds. As our previous metabolite screening study had higher abundances of selected polyphenolic compounds in the red ecotype [9], our hypothesis for this study was that *D. dentatus*’ red ecotype would have higher endogenous CK levels when compared to the white ecotype. To date, no prior instances of CK profiling in Dilleniaceae, especially for *D. dentatus*, have been reported. Although we previously reported on the phytochemical profiling of *D. dentatus* via mass spectrometry-based untargeted metabolomics [9], we now present evidence that *D. dentatus* ecotypes contain different CK forms that may be able to influence its wider phytochemical profiles. By explaining these relationships, we hope to gain insights into the regulation of complex biochemical processes and signalling pathways involved in secondary metabolite production. Ultimately, this study aims to deepen our understanding of the therapeutic potential of *D. dentatus*’ ecotypes.

## 2. Materials and Methods

### 2.1. Collection Sites for D. dentatus’ Red and White Ecotypes—Eagle Mountain Forest Potaro—Siparuni, Guyana

**Figure 3:**
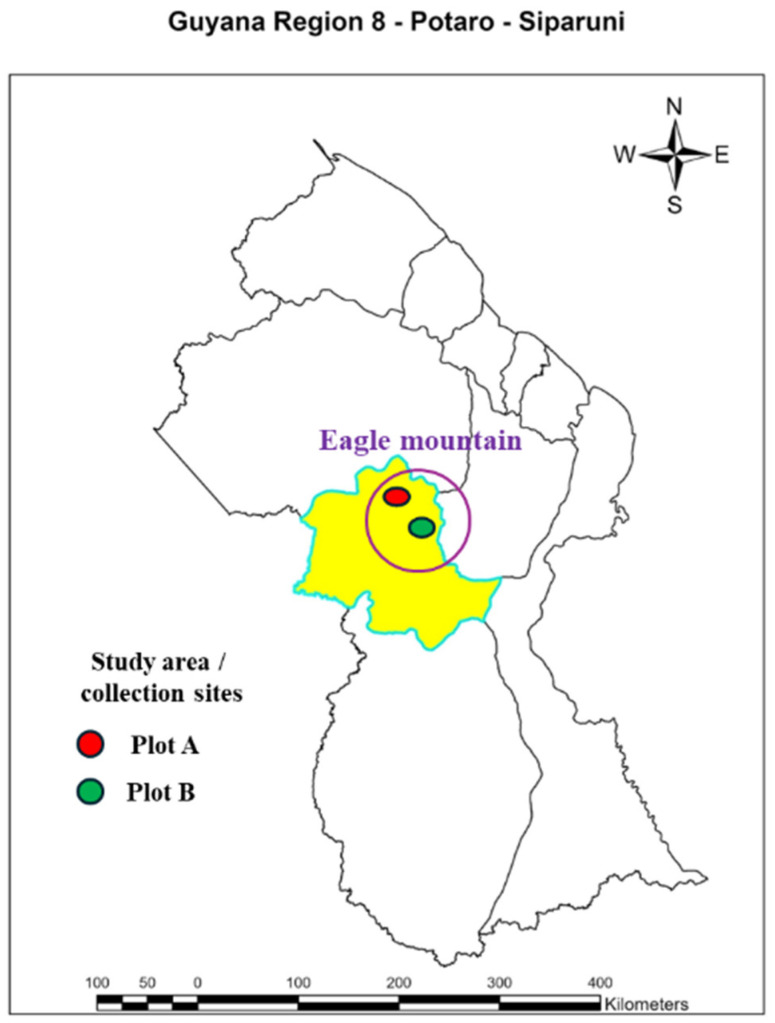
Map of the Study area on the Eagle Mountain Forest located in Potaro Siparuni (21N0261909-0578704) Region 8. The yellow area highlights Potaro Siparuni Region 8 of which the study area is a part of. Plot A the collection of the *D. dentatus* red ecotype represented by a red circle. Plot B collection of *D. dentatus* white ecotype represented by the green circle.

In July 2022 and December 2024, a total of three biological samples of the vine of the red and white ecotypes were randomly harvested from the *D. dentatus* liana population found in the Eagle Mountain Forest (Figure 3). The vine (i.e., liana), being a vascular plant comprises the phloem and xylem [88] that is ground. A biological sample refers to plant material collected from the same wood vine [89]. *D. dentatus*’ red ecotype was collected in plot A, 200 m from plot B where *D. dentatus*’ white ecotype was collected. These collections included discs of both red and white *D. dentatus*, ranging in diameter classes from 4 cm to 20 cm [9]. The differences between *D. dentatus* red and white are recognized by their wood colour (Figure 1) instead of their botanical features such as leaves, outer and inner barks and fertile organs [8]. Only the vine was used for this study, similarly to the work published previously [9].

### 2.2. Preparing Biological Samples and Extracting and Purifying Phytohormones and Other Secondary Metabolites

**Figure 4:**
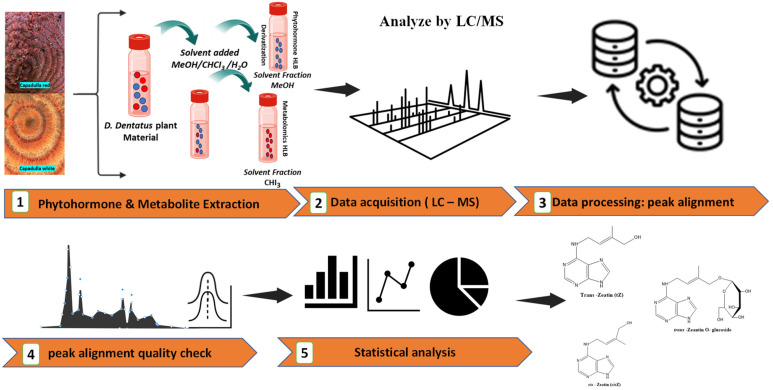
The workflow diagram outlines a detailed analysis process for phytohormones and untargeted metabolites in *D. dentatus* plant material. The orange arrows present the different steps in the analysis. (**1**) the extraction using a MeOH/CHCl_3_/H_2_O solvent mixture, resulting in separated fractions. (**2**–**3**) LC-MS analysis flow, with data processing focused on peak alignment and quality control through chromatographic profiles. (**4**) the statistical analysis and visualization using bar charts, line graphs, and pie charts. (**5**) the final output identifies specific phytohormones and secondary metabolites compounds. The icons used are a combination of Noun Project and BioRender.com.

Multihormone and metabolite extractions were performed with modifications to previously published protocols [33,90,91,92,93,94,95,96]. To give rise to Guyana Capadulla extract (GCE) samples (i.e., Figure 1A–D), the woody stems of *D. dentatus*’ ecotypes were pulverized using a Thomas Wiley Mill (Model ED-5; Greater Minneapolis, Minneapolis, MN, USA). Stainless steel grinding cylinders were used for tissue (xylem and phloem) greater than ~0.25 g (25 mZ, 30 s, 4 °C, Retsch MM300; Haan, Germany). The ground samples were sieved (0.0613 mm size sieve) and immediately stored at −80 °C for later extraction. Thirty (30 ± 0.03) mg of plant tissue was weighed out in round bottom 2 mL centrifuge tubes and spiked with 10 nanograms (ng) of each internal standard (IS; Figure 4; Table 1 and Table 2) on ice. In each sample, 1 mL of 80% methanol (MeOH), 0.5 mL of chloroform (CHCI_3_), and 0.2 mL of water (ddH_2_O) were added. Two zirconium oxide grinding beads (Comeau Technique Ltd., Vaudreuil-Dorion, Canada) were placed in each sample and homogenized using a Retsch MM300 ball mill (Haan, Germany) at 25 Hz for 5 min in a 4 °C room.

Homogenized GCE samples were sonicated, vortexed and centrifuged at 8400× *g* for 20 min (ThermoScientific Sorvall ST 16 Centrifuge). Using MeOH, CHCI_3_ and ddH_2_O resulted in a phase separation into polar and nonpolar fractions [96]. The polar fraction on the top layer was decanted into another vial (Figure 4). A second round of extractions for the polar fraction was performed in the same way, except residues were reconstituted in 1 mL of 1 M formic acid to completely protonate all CKs [33,97]. Table 1 and Table 2 contain a list of all the acidic and CK phytohormones scanned that were detected with their internal standards and others scanned for but not detected.

For sample cleanup, solid phase extraction (SPE) was performed using HLB cartridges (VIOLET™ 200 mg/6 mL, 40 mL; Canadian Life Sciences; Peterborough, ON, Canada), which were pre-conditioned with methanol, ddH_2_O, and 50% ACN before loading GCE supernatants. Samples were loaded onto HLB cartridges, and allowed to elute via gravity, then 2 mL of 30% aqueous ACN was added on top of each sample. Afterwards, each collected extract was divided into two fractions: one for CK analysis, and the other for derivatizing acidic phytohormones containing other metabolites. All samples were evaporated to dryness at ambient temperature in a speed vacuum centrifuge concentrator (Thermo Savant UVS 400a; ThermoFisher Scientific, Berlin, Germany).

The chloroform fraction samples were not subjected to HLB, nor were derivatized, but were dried and resuspended, centrifuged and stored at −20 °C for mass spectrometry analysis.

### 2.3. Solid Phase Extraction of CKs

CK extraction was performed via a modified published protocol [33,87,97]. Previously dried supernatant residues were reconstituted in 1 mL of 1 M formic acid (HCOOH) to completely protonate CKs. The reconstituted samples were subjected to SPE using MCX cartridges (Oasis MCX 6 cc; Waters, Mississauga, ON, Canada), specific for polar compounds like CKs [56]. Prior to eluting, the cartridges were activated with 5 mL of methanol and equilibrated with 5 mL of 1 M formic acid. After cartridge equilibration, samples were loaded onto cartridges, allowed to elute by gravity, and subsequently washed with 5 mL of 1 M formic acid [33,87,97].

Collected samples were dried and then resuspended in 300 μL of acetic acid (AcOH): acetonitrile (ACN): deionized water (ddH_2_O) in a volumetric ratio of 0.08:5:94.92 (v:v:v), respectively. Samples were then centrifuged at 8400× *g* for 10 min and the supernatants transferred to 2 mL clear vials fitted with 350 μL glass inserts and stored at −20 °C until mass spectrometry analysis.

### 2.4. Derivatizing Acidic Phytohormones

Phytohormone derivatization was based off a slightly modified published protocol [91,97]. Briefly, dried samples were reconstituted with 75 μL of 1-propanol, 20 μL of ddH_2_O, 5 μL of 500 mM bromocholine in 70% ACN, and 1 μL of triethylamine, then vortexed and incubated in a hot water bath at 80 °C for 130 min. Following incubation, samples were ice cooled then dried using a speed vacuum concentrator (Thermo Savant UVS 400a; ThermoFisher Scientific, Berlin, Germany) at ambient temperature for 3 h [91,97]. Dried samples were resuspended and stored for mass spectrometry analysis, as indicated above. Derivatization reagents were sourced from Fisher Scientific (Ottawa, ON, Canada).

### 2.5. Acquiring Phytohormones and Secondary Metabolites Data Using Liquid Chromatography-Mass Spectrometry

The LC-MS gradient and mass spectrometry acquisition methods used were as previously published [9,33,36] using a Thermo Q-Exactive™ Orbitrap mass spectrometer, coupled to a Thermo Dionex Ultimate 3000 Liquid Chromatograph System (Thermo Scientific, San Jose, CA, USA). CKs and acidic phytohormones were monitored using a full scan in positive ionization mode for 10 min. A parallel set of samples was split off from the CK profiling samples described above for red and white ecotypes and these underwent untargeted metabolomics analysis (Figure 4). For untargeted metabolomics, metabolites in the methanol (MeOH) and chloroform (CHCl_3_) fractions were analyzed using mixed ionization modes (i.e., polarity switching of positive and negative modes) for 10 min. The mass range scanned for phytohormones, and metabolites was from 80 to 900 *m*/*z*, and the resolution was 140,000 at 200 *m*/*z* full width at half maximum (FWHM). The automatic gain control (AGC) target was set to 3 × 10^6^, and the maximum injection time (IT) was 524 ms. For the heated electrospray ionization (HESI) probe, the capillary temperature was set to 250 °C, the sheath gas was 30 arbitrary units, and the auxiliary gas was 8 units. The probe heater temperature was set to 450 °C, the S-Lens RF level was 60%, and the capillary voltage was 3.9 kV.

Data dependent acquisition (DDA; ddMS^2^) was performed in positive and negative ionization modes within a scan range of 80 to 900 *m*/*z* at a resolution of 17,500 with an AGC target of 1 × 10^6^, using a representative sample from each fraction (i.e., MeOH or chloroform). The fragmentation was triggered at a loop count of 10, with a precursor isolation window of 1 *m*/*z*, and at a normalized collision energy (NCE) of 30. The maximum IT was 64 ms.

Phytohormones and other metabolites were separated using a multistep gradient as follows: mobile phase B was held at 0% for 30 s, before increasing it to 100% over 3 min. Solvent B was then held at 100% for 2 min before returning to 0% over 4 min for column re-equilibration [9]. Injection volume was 25 μL. Chromeleon 6.8 Chromatography Data System software (Thermo Scientific; Ottawa, Canada) was used to control the instrument.

### 2.6. Quantifying Endogenous Phytohormone Levels Using Isotopic Dilution Analysis

Phytohormone categorization based on CK groups (i.e., aromatics, free bases, ribosides, glucosides, and methylthiols) or CK type classification (i.e., iP, *t*Z, *c*Z, and DZ types) are specified in Table 1 and Table 2. However, as CK nucleotide forms cannot be detected with our LC-MS methodology, we report on free base, riboside, and glucoside CK forms.

Isotope dilution analysis was used to determine endogenous phytohormone levels by quantifying the peak areas of the endogenous analytes relative to those of internal standards (IS; [33]). Phytohormone levels were calculated using the following formula:

$$\mathrm{Phytohormone}\mathrm{concentration}\left[\frac{pmol}{g}DW\right]=\frac{\frac{Analyte\;peak\;area}{IS\;peak\;area}*\frac{IS\;mass}{\left(MW\;of\;IS\;*\;1000\right)}}{mass\;of\;plant\;tissue}$$

where DW − mass of the tissue, mass of IS = (10 ng, 20 ng or 60.1 ng for CKs, GAs and ABA, respectively; Table 1 and Table 2) as per used in method; MW − molecular mass of each CK, or other acidic phytohormones [g] [33]. Three biological replicates (*n* = 3) of each plant sample ecotype were analyzed. All detected phytohormones were at Metabolomics Standard Initiative (MSI) level 1 annotation [98,99].

### 2.7. Metabolomics Analysis of D. dentatus’ Ecotypes: Data Processing of Untargeted Mass Spectrometry Data

ProteoWizard’s MSConvert module was used to convert mass spectrometry data from *. RAW to *.mzXML format (Figure 4; [100,101,102]). Although LC-MS data acquisition was performed in mixed ionization full scan mode, negative and positive ions were extracted separately for metabolomic analysis using the subset filter option in MSConvert.

Parameters used for metabolomics analyses were as previously published [9]. Briefly, *.mzXML files were uploaded to XCMS Online [103,104], for peak identification, retention duration adjustment, and grouping. The chemical composition of *D. dentatus*’ red and white ecotypes was analyzed using various databases and analytical tools within a ±5 ppm error margin, such as MetaboQuest (created from MetaboSearch; [105]), PubChem [106], the Plant Metabolic Network (i.e., PMN via PlantCyc database; [107]), KNapSAcK [108], SciFinder (https://scifinder.cas.org, accessed on 15 April 2024) (These databases and tools were accessed on 15 April 2024), KEGG [109] and MetaboAnalyst 6.0 [110] were used to check for putative metabolites as previously published [9]. These databases and tools were accessed on 15 April 2024. Compound comparisons per ecotype with matches from databases for physiologically active substances, and potential medicinal chemical families (i.e., flavonoids, terpenoids, and alkaloids) were inputted into ThermoScientific XCalibur software (v. 4.1) to create a method for peak identification via semi-quantification using relative intensities based on protonated and deprotonated masses [9,97].

### 2.8. Statistical Analysis of Metabolomics Datasets

Statistical analysis was performed on XCMS derived parsed data using MetaboAnalyst v. 6.0 (https://www.metaboanalyst.ca, accessed on 15 April 2024), with normalization, log transformation, PCA, and PLS-DA for visualization [110]. Data normalization was executed using sum normalization, log_2_ transformation, and Pareto scaling [110]. These pre-processing steps were critical for data calibration to a common scale for equitable comparison and robust statistical evaluation. After data rescaling and normalization, ANOVA and volcano plot analyses were used to identify any differences between red and white ecotypes and to identify *m*/*z* features that had changed significantly (*p* < 0.05). A standard volcano plot was used to identify different metabolites using t-tests and fold-change methods [111]. This plot depicted log-transformed fold-change values against negative log-transformed *p*-values from t-tests. Analysis of Variance (ANOVA) and comparison of Least Significant Difference (LSD) were applied to compare associations between CKs and alkaloids, flavonoids, terpenoids and other compounds present in *D. dentatus* ecotypes.

### 2.9. Processing Untargeted Tandem Mass Spectrometry (MS^2^) Data Acquired via DDA

mzMine (v. 2.5.3) was utilized to match MS^1^ features corresponding to MS^2^ fragments, involving mass detection, chromatogram construction, smoothing, deconvolution, deisotoping, alignment, and gap filling [9,97,112,113,114,115]. Data were stored in *.mgf format.

MS-DIAL v. 5.5 [116], the feature based molecular networking and classical molecular networking modules of GNPS [117,118], SIRIUS v. 5.6.3 [119], and MS2Compound v. 1.03 [120] were used to identify compounds based on matched fragments, using previously published workflows [9,97]. Following the recommendations of the Metabolomics Standards Initiative (MSI), metabolite annotation for MS-DIAL and GNPS were at MSI level 2, while SIRIUS and MS2Compound were at MSI level 3 [98,99]. Cheminformatics for grouping compound annotations (from MS-DIAL, GNPS, SIRIUS, MS2Compound and semi-targeted analysis) into different compound classes were performed as previously published [9,97,121]. In addition, the Chemical Translation Service [122] was used to standardize metabolite information.

Job IDs/links for GNPS are as follows (created between 4 to 7 February 2025):

**Classical based molecular networking**

1. Chloroform fraction in positive ionization mode:
  https://gnps.ucsd.edu/ProteoSAFe/status.jsp?task=645b50127491458eaadbc9b09a7f7886 (accessed on 15 April 2024)
2. Chloroform fraction in negative ionization mode:
  https://gnps.ucsd.edu/ProteoSAFe/status.jsp?task=43657c1864c948beaf7c3be4655f1490 (accessed on 15 April 2024)
3. Methanol (HLB) fraction in positive ionization mode:
  https://gnps.ucsd.edu/ProteoSAFe/status.jsp?task=bb377d2cabed43ecb62f53bbf35b4fa0 (accessed on 15 April 2024)
4. Methanol (HLB) fraction in negative ionization mode:
  https://gnps.ucsd.edu/ProteoSAFe/status.jsp?task=74499f8df56e483eaf664cd803425fef (accessed on 15 April 2024)

**Feature based molecular networking**

1. Positive ionization mode:
  https://gnps.ucsd.edu/ProteoSAFe/status.jsp?task=afdf9d79c69947b590417566d49d8b9a (accessed on 15 April 2024)
2. Negative ionization mode:
  https://gnps.ucsd.edu/ProteoSAFe/status.jsp?task=be589f20e69245188f0b9bb379606c40 (accessed on 15 April 2024)

### 2.10. Correlation Analysis of Endogenous Phytohormones and Select Secondary Metabolites

Metabolite AutoPlotter v2.6 is an analytical tool designed for analyzing and visualizing metabolomic data, encompassing correlation analysis [123]. Data was parsed into excel files, specifically with designated columns for phytohormones and metabolites. Upon uploading the data, specific variables (i.e., ecotypes, compounds, CKs and *p*-values based on the correlation coefficients) were analyzed to assess correlations. The Pearson correlation coefficients served as the statistical measure for evaluating the associations between CKs and different classes of secondary metabolites (i.e., flavonoids, terpenoids and alkaloids). For visualization purposes, heatmaps and network diagrams were utilized. These visualizations are customizable, allowing for the accentuation of strong correlations or the highlighting of specific patterns. The default settings were employed to identify significant correlations based on coefficient values and *p*-values. Ultimately, the correlation matrix and accompanying visualizations were exported for subsequent analysis. Further analysis was conducted on the data processed using Metabolite AutoPlotter v2.6 (https://mpietzke.shinyapps.io/autoplotter; accessed on 15 April 2024) in conjunction with R Studio 12.0 [124] to confirm and validate data.

## 3. Results

### 3.1. Phytohormone Profiling and Metabolome Analysis of D. dentatus’ Red and White Ecotypes

For *D. dentatus*’ red and white ecotypes, phytohormone profiles were conducted via a targeted metabolomics approach, while a semi-targeted approach was adopted for the metabolomic analysis via LC-MS. Putative *m*/*z* features identified in the metabolomics study were compared against databases containing physiologically active compounds documented in the scientific literature, and widely used bioinformatics servers to distinguish metabolite features, and GNPS, MS-DIAL, and SIRIUS for confirming fragments from DDA (Tables S1–S6; workflow as previously published [9]).

**Figure 5:**
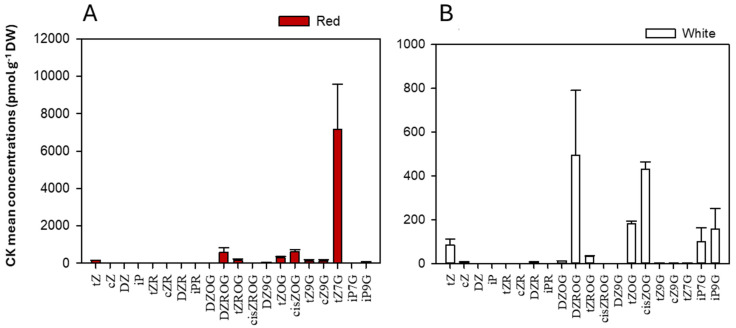
The mean CK concentrations and forms (glucosides, freebase and ribosides) (pmol*g^−1^ DW) in *D. dentatus* ecotypes. (**A**) CK concentrations in *D. dentatus*’ red ecotype. (**B**) CK concentrations in *D. dentatus*’ white ecotype. Data shown represents a sum of total CK content from both chloroform and methanol (HLB) fractions.

Using targeted metabolomics, 35 different CK forms in plant tissues were scanned (Table 1, Table 2 and Table S1), with 20 CKs detected (Figure 5; Table 1 and Table 2), and classified into free bases, ribosides, and glucosides (Figure 5; Table 3). Among these, CK glucosides, considered mostly inactive [48,125,126,127,128,129], were identified in *D. dentatus* ecotypes, including DZOG, DZROG, DZ9G, *t*ZOG, *t*ZROG, *t*Z9G, *c*ZOG, *c*ZROG, and *c*Z9G (Table 3). In addition, active CK forms (i.e., iP, *c*Z, *t*Z and DZ) and their immediate riboside conjugates (i.e., iPR, cZR, *t*ZR and DZR) were detected Figure 5 and Figure S1, Table 1 and Table 3). *D. dentatus*’ red ecotype showed higher levels of most CKs, particularly in the glucoside form (98.38%; Figure 5, Table 3). The most notable exceptions were iP7G and iP9G, shown to be higher in the white ecotype. Overall, the red ecotype displayed higher free base levels (1.83%). The red ecotype exhibited a substantially higher total CK content (9135.49 ± 3174.72 pmol*g^−1^ DW) when compared to the white ecotype (1478.53 ± 527.21 pmol*g^−1^ DW), indicating a marked difference in CK accumulation between these two ecotypes.

**Figure 6:**
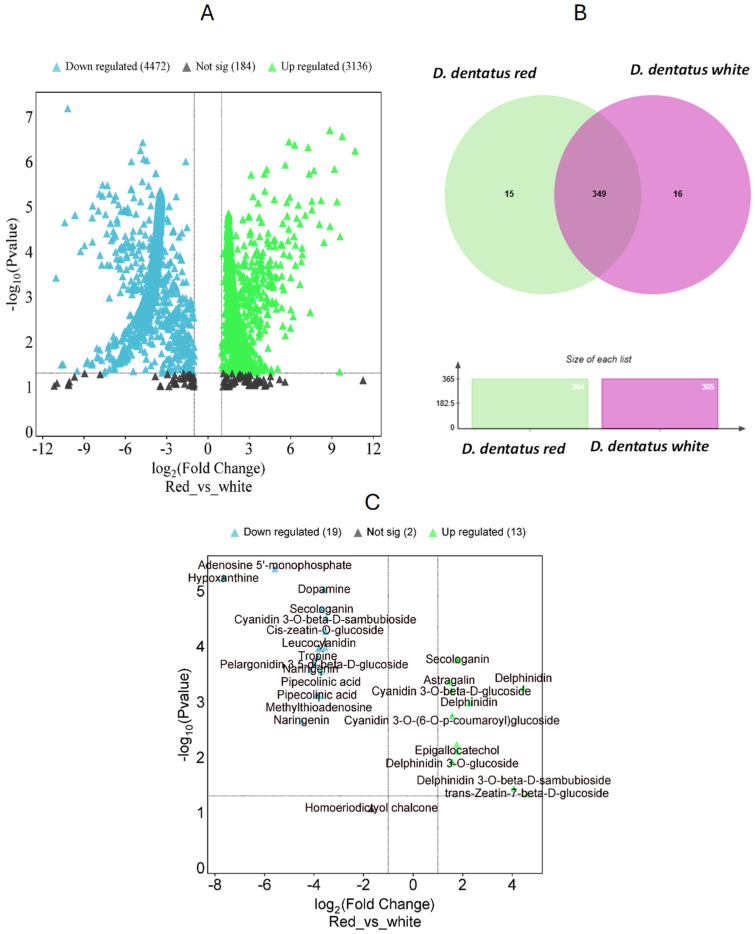
Comparative analysis of phytochemical profiles between *D. dentatus* red and white ecotypes. (**A**) Volcano plot displaying differential putative metabolites between red and white ecotypes, where green indicates upregulated metabolites in red, blue indicates downregulated metabolites, and black represents non-significant changes. The *x*-axis corresponds to the logarithmic fold change, which indicates the extent and direction of tentative metabolite expression changes. The *y*-axis provides the −logarithmic *p*-values, which indicate the statistical significance of these changes. (**B**) Venn diagram highlighting shared and unique metabolites in red and white ecotypes, accompanied by bar plots showing the total number of metabolites identified in each ecotype. (**C**) Highlighted metabolites with significant fold changes, showing key bioactive compounds such as secondary metabolites and phytohormones with differential abundance between the two ecotypes.

Analyzing *D. dentatus* extracts revealed significant differences in metabolite profiles in the *D. dentatus*’ red and white ecotypes. The red ecotype contained 20,382 potential metabolite features both positive and negative ionization modes. In contrast, the *D. dentatus* white ecotype exhibited 11,021 potential *m*/*z* features in both positive and negative ionization modes (Figure 6). Further analysis of the metabolomic data using ANOVA depicted in the volcano plots (Figure 6 and Figure S2), showed 5275 *m*/*z* tentative metabolite features were identified in the red ecotype, with 2829 *m*/*z* in the positive ionization mode. In *D. dentatus*’ red ecotype, 159 *m*/*z* features were upregulated, and 110 *m*/*z* features were downregulated, using the white ecotype as a control to identify differences in metabolite profiles. These findings aligned with previous research, which reported higher upregulation of polyphenols and flavonoids (including flavones, flavanones, flavanonols, and flavone-3-ols) in the red ecotype compared to the white ecotype [9].

In the negative and positive ionization modes, 2446 *m*/*z* tentative features were analyzed, resulting in 98 *m*/*z* features being upregulated and 88 *m*/*z* features downregulated in the *D. dentatus* red ecotype. To further our understanding of the metabolic differences between the two ecotypes, we utilized the “Functional Analysis” module and the Gene Set Enrichment Assay (GSEA) tool in MetaboAnalyst 6.0 (Figure 6 and Figure S2). We compared the 5275 potential metabolite characteristics acquired from examining the two ecotypes in combined fractions. Utilizing the GSEA program, we searched inside the annotated *A. thaliana* metabolite database to find metabolite characteristics that closely corresponded to our *m*/*z* features. This allowed us to recognize resemblances with the Kyoto Encyclopedia of Genes and Genomes (KEGG) database and GNPS, MS-DIAL, and SIRIUS (as seen in the workflow previously published [9]) services in untargeted metabolomics at MSI levels 2 and 3 [98,99].

Upon completion of the global metabolomic analysis, we found 349 putative metabolite features with annotation at level 3 based on matching reference databases. Among these, 16 features (0.9%) were exclusive to the *D. dentatus* red ecotype (e.g., kaempferol and luteolin), while 17 putative features (e.g., dihydromyricetin, eriodyctiol and eriodyctiol chalcone) belong to the *D. dentatus* white ecotype (4.2%). Notably, approximately (91.5%) of these putative metabolite features, were common to the *D. dentatus* red or white ecotypes (Figure 6).

**Figure 7:**
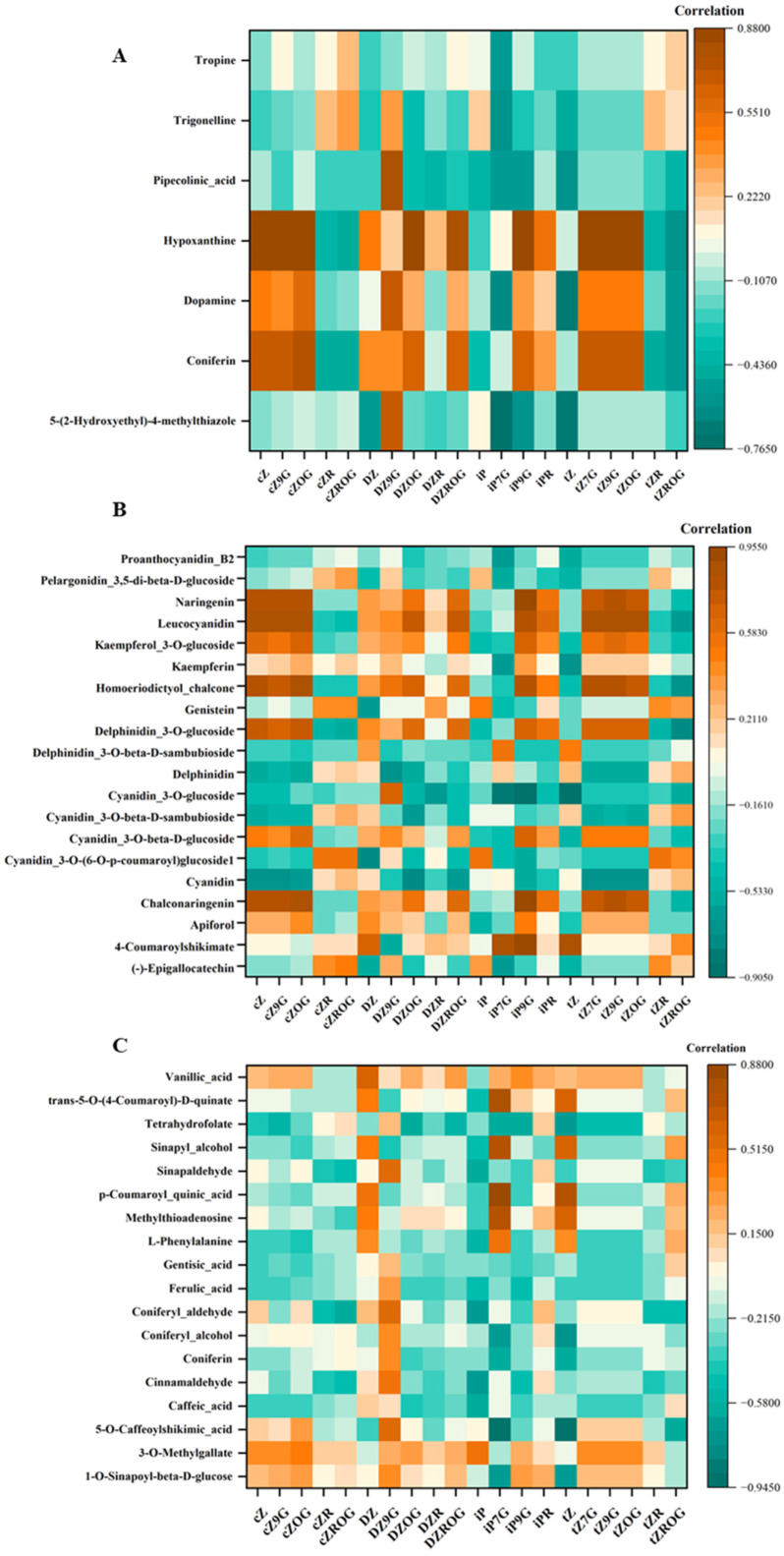
Correlation heatmaps showing the relationships between CKs and (**A**): selected alkaloids, (**B**): selected flavonoids, and (**C**): selected phenolic compounds in *D. dentatus* ecotypes. Brown colors indicate positive correlations while green colors represent negative correlations.

MetaboQuest confirmed our matches of the putative *m*/*z* from the previous search (Figure 6). Putative features, searched within a ±5 ppm error range, were at MSI level 3 identification using MetaboAnalyst 6.0 and associated with KEGG pathways. Using XCalibur, a method was created to verify the masses of matched flavonoids, terpenoids, and alkaloids in sample replicates [9]. Compounds such as naringenin, delphinidin, delphinidin 3-O-glucoside, *trans*-Zeatin-beta-D-sambubioside, epigallocatechol, and cyanidin 3-O-beta-D–sambubioside, among others were matched (Figure 7 and Figure S2–S7; Tables S2–S6). *D. dentatus* ecotypes were previously reported to be dominated by polyphenols (63.9% contribution in the phenylpropanoid compound class as previously published [9]). Polyphenols are essential bioactive phytochemicals present in many plant species, renowned for their powerful antioxidant activities. Considering these benefits, we undertook a more comprehensive investigation of compounds under the polyphenol class, within the *D. dentatus* red ecotype.

### 3.2. Integration of Correlation Network Analysis with CKs and Secondary Metabolites: Alkaloids, Flavonoids and Other Phenolic Compounds

**Figure 8:**
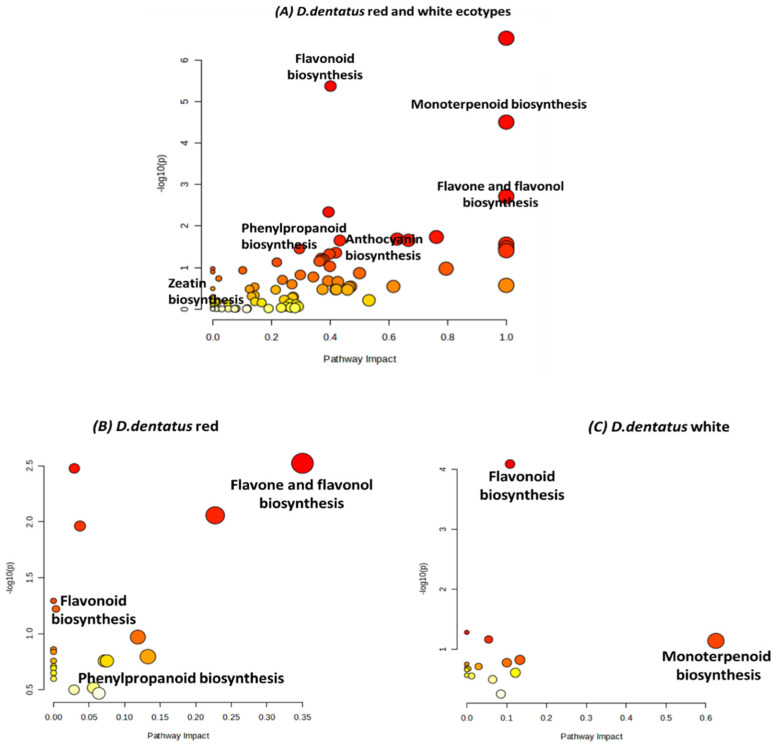
Pathway enrichment analysis comparing metabolic pathways in red and white ecotypes of *D. dentatus* pathways obtained from the KEGG and HMDB. Each dot represents a specific metabolic pathway, with the size of the dot indicating pathway impact and its colour intensity reflecting significance (red = highly significant; yellow = less significant). The *x*-axis (Pathway Impact) quantifies the influence of each pathway based on metabolite contributions, while the *y*-axis (−log10(p)) denotes statistical significance. These *p*-values are obtained from the functional analysis pathway enrichment.

The study utilized correlation network analysis to find possible associations between CKs and secondary metabolites such as alkaloids, flavonoids and other phenolic compounds in *D. dentatus*’ ecotypes (Figure 7, Tables S7–S9). The correlation matrix revealed relationships between Zeatin derivatives and other compounds, where orange indicated positive correlations and teal indicated negative correlations (Figure 7). *t*Z and iPR CKs exhibited significant interrelationships with various secondary metabolites, including alkaloids, phenolics, and flavonoids. Notably, *t*Z and iPR demonstrated a strong positive correlation with phenolic compounds, particularly 1-O-sinapoyl-beta-D-glucose. Additionally, tropine revealed a strong positive correlation with *c*Z and its derivatives within the alkaloid category. Furthermore, flavonoid analysis indicated that leucocyanidin was strongly associated with several types of cytokinins, especially *t*Z derivatives (Figure 7). The red ecotype also exhibited enrichment in the flavonoid biosynthesis pathway (Figure 8).

The correlation matrix revealed relationships between different Zeatin forms and other compounds, where orange indicated positive correlations and teal indicated negative correlations (Figure 7). *c*Z type CKs (i.e., cZ, *c*Z9G, *c*ZOG, *c*ZR, and *c*ZROG) showed positive correlations with coniferin and dopamine, while *c*Z, *c*Z9G, *c*ZR, and *c*ZROG exhibited negative correlations with pipecolinic acid (Figure 7). DZ type CKs (i.e., DZ, DZR, DZ9G, DZOG, and DZROG) were positively correlated with hypoxanthine and coniferin and negatively correlated with pipecolinic acid. iP type CKs (i.e., iP, iP7G, iP9G, iPR) were positively correlated with hypoxanthine and dopamine. Finally, *t*Z type CKs (i.e., tZ, *t*Z7G, *t*Z9G, *t*ZOG, *t*ZR, and *t*ZROG) had positive correlations with tropine and negative correlations with coniferin. Proanthocyanidin B2, pelargonidin 3,5-di-beta-D-glucoside, naringenin, and leucocyanidin showed strong positive correlations among themselves, but negative correlations with *t*Z type CKs (i.e., tZ, *t*Z7G, *t*Z9G, *t*ZOG, *t*ZR, and *t*ZROG). Kaempferol 3-O-glucoside, kaempferin, and homoeriodictyol chalcone were positively correlated, but were negatively correlated with *c*Z type CKs (i.e., cZ, *c*Z9G, *c*ZOG, *c*ZR, and *c*ZROG). *t*Z was positively correlated with delphinidin 3-O-glucoside, which in turn showed positive correlations with delphinidin 3-O-beta-D-sambubioside and delphinidin. Further positive correlations were observed within the cyanidin series (cyanidin 3-O-glucoside, cyanidin 3-O-beta-D-sambubioside, cyanidin 3-O-beta-D-glucoside, and cyanidin 3-O-(6-O-p-coumaroyl) glucoside), as well as between cyanidin, chalconaringenin, apiforol, 4-coumaroylshikimate, and (-)-epigallocatechin. Notably, (-)-epigallocatechin exhibited positive correlation with *c*Z type CKs (i.e., cZ, *c*Z9G, *c*ZOG, *c*ZR, and *c*ZROG), while DZ type CKs (DZ, DZ9G, DZOG, DZR, DZROG) showed some correlations with other compounds, and *tZ* derivatives were negatively correlated with proanthocyanidin B2, pelargonidin 3,5-di-beta-D-glucoside, naringenin, and leucocyanidin (Figure 7).

The correlation matrix displayed relationships between Zeatin derivatives and other compounds, with orange indicating positive and teal indicating negative correlations. *c*Z type CKs like *c*Z, *c*Z9G, *c*ZOG, *c*ZR, and *c*ZROG showed positive correlations with 3-O-methylgallate, with *c*ZOG also positively correlated with sinapyl alcohol, while they generally exhibited negative correlations with vanillic acid. DZ type CKs (i.e., DZ, DZ9G, DZOG, DZR, and DZROG) were positively correlated with coniferyl alcohol and 3-O-methylgallate, with DZOG also showing a positive correlation with sinapyl alcohol, and these forms also showed negative correlations with vanillic acid. In contrast, *t*Z type CKs (i.e., tZ, *t*Z7G, *t*Z9G, *t*ZOG, *t*ZR, and *t*ZROG) had positive correlations with sinapyl alcohol and negatively correlated with 5-O-caffeoylshikimic acid, with *t*Z7G and *t*Z9G showing weak correlations overall.

### 3.3. Visualizing Metabolome Diversity: A Compelling Analysis of Biosynthetic Signatures in Red and White D. dentatus Ecotypes

Pathway enrichment analysis with pathway impact values combines *p*-values in a topology analysis using KEGG pathways in MetaboAnalyst 6.0 in the form of a scatter plot. Scatter plot data revealed the impacts and significance of various biosynthesis pathways in *D. dentatus* ecotypes (Figure 8). In the red ecotype, key pathways such as flavonoid biosynthesis, monoterpenoid biosynthesis, flavone and flavonol biosynthesis showed high impact and significance, as evidenced by large, dark red dots. These pathways are closely associated with secondary metabolites involved in pigmentation and aroma, reflecting the red ecotype’s distinct colouration. Additionally, anthocyanin biosynthesis, another pathway related to pigmentation, displays significance (*p* = 0.02 < 0.05), further highlighting the metabolic emphasis on colouration in the red ecotype.

In contrast, the white ecotype exhibited enrichment in pathways such as phenylpropanoid biosynthesis and flavonoid biosynthesis, but these showed lower pathway impact and significance compared to the red ecotype. Flavone and flavonol biosynthesis were less prominent, reflecting reduced metabolic activity in pigmentation-related pathways. Furthermore, monoterpenoid biosynthesis, significant in the red ecotype, showed a minimal impact in the white ecotype (Figure 8). These differences indicate that the red ecotype is metabolically geared towards pigmentation and aroma biosynthesis, while the white ecotype demonstrates lower activity in these pathways, consistent with its lack of pigmentation.

### 3.4. Using ClassyFire to Highlight the Chemodiversity of Annotated Compounds in D. dentatus

**Figure 9:**
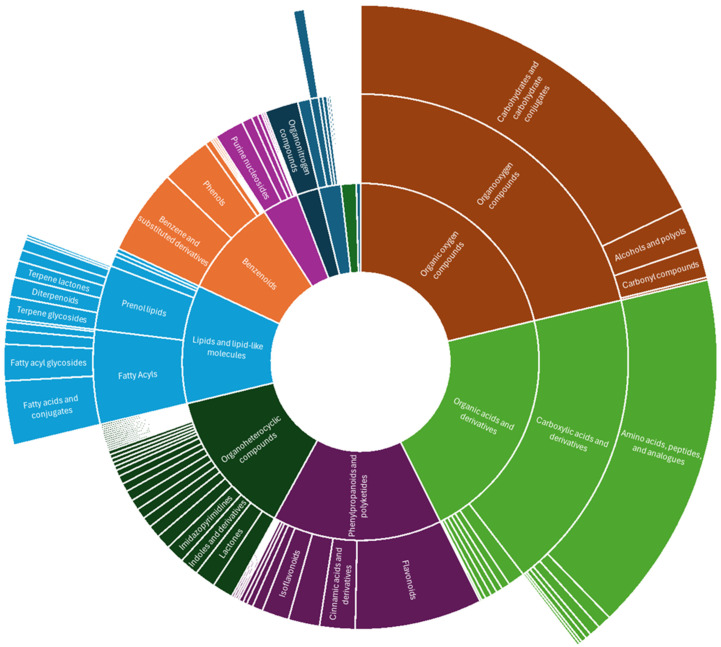
Sunburst plot showing annotated compounds classified into compound classes via ClassyFire from both positive and negative ionization modes from red and white ecotypes of *D. dentatus*.

Based on the categorization of compounds via ClassyFire [121] as represented by the sunburst plot (Figure 9), 794 compounds at MSI annotation levels 1 to 3 in *Doliocarpus dentatus* were mostly grouped under phenylpropanoids and polyketides (14.9%), organic acids and derivatives (21.5%), organic oxygen compounds (21.3%), lipids and lipid-like molecules (10.7%) and organoheterocyclic compounds (13.3%) (Figure 9). This constitutes both ecotypes with compounds extracted from both chloroform and methanol fractions. For the compound class of lipids and lipid-like compounds, terpenoids, which falls under prenol lipids, accounted for 35.7% (Figure 9). Flavonoids, which falls under phenylpropanoid and polyketides compound class (also including isoflavonoids and other flavonoid types), constituted 61.5% (Figure 9). Alkaloids and derivatives accounted for 2% of annotated compounds. Surprisingly, carboxylic acids and derivatives constituted most organic acids and derivatives (85.7%), of which amino acids, peptides and analogues dominated (90.3%) (Figure 9). This shows that despite differences in extraction methods previously published compared to these results, there is still representation for flavonoid and terpenoid biosynthesis.

## 4. Discussion

### 4.1. Phytohormone Profiling of D. dentatus’ Ecotypes Reveal Contrasting CK Concentrations

*D. dentatus*’ red and white ecotypes demonstrated substantial medicinal potential due to their distinct phytochemical profiles, particularly in their CK profile and secondary metabolite composition. *D. dentatus*’ extracts were rich in bioactive compounds such as alkaloids, phenolics, flavonoids, and triterpenes (Figure 7, Table 3; [9]) which may contribute to its potential therapeutic properties, including anti-inflammatory, antioxidant, antimicrobial, antitumor and antidiabetic effects [125,126,127,128,130,131]. CKs in *D. dentatus*, such as *t*Z cZ and iPR, have shown promise in reducing oxidative stress, a factor in chronic diseases such as cancer and neurodegenerative disorders [51,132,133]. Traditional use of *D. dentatus* for conditions such as arthritis, diabetes, and gastrointestinal disorders supports its therapeutic potential, with the red ecotype showing higher CK concentrations and enhanced bioactivity compared to the white ecotype [13,25,130,131,134,135,136].

CKs, particularly those in the red ecotype, offer potential therapeutic benefits, including antioxidant, anti-ageing, and neuroprotective effects [137,138,139,140]. Elevated levels of CKs such as *t*Z, among other Zeatin types and its derivatives in glucoside forms (i.e., DZOG, DZROG, DZ9G, *t*ZOG, *t*ZROG, *t*Z9G, cZOG, cZROG, and cZ9G) in the red ecotype suggest improved storage, transport, and activation mechanisms [11,73,74]. Ribosides such as DZR (Figure 5, Table 3) play crucial roles in CK metabolism and could contribute to prolonged therapeutic efficacy when extracted for human applications. Controlled activation of CK conjugates, such as *t*Z7G and *t*Z9G, may enable sustained-release systems, reduce side effects and enhance stability in targeted treatments [85,141,142,143,144]. This could be particularly useful in areas such as cancer therapy, immune modulation, and wound healing [136,144,145], where the red ecotype’s enhanced CK profile could provide distinct advantages.

The presence of O-glucosides and N-glucosides in *D. dentatus* further emphasizes the adaptability and potential therapeutic relevance of this species. In plants, glycosylation (i.e., adding of a carbohydrate moiety) is thought to deactivate CKs via the uridine 5′-diphospho-glucuronosyltransferase (UGT) enzyme acting at the O- or N-position of the CK molecule (Figure 2; [128,146]). Although N-glucosides form especially when CK levels are high due to gene overexpression or external CK application and were believed to be irreversible products, O-glucosides, when de-glycosylated, can act as a storage form of free-CKs [128,146]. Large amounts of N-glucosides of isopentenyl adenine (iP) and *trans*-Zeatin (*t*Z) are found outside plant cells, particularly in the xylem [147]. For quite some time, N-glucosylation of cytokinins were considered irreversible. However, *t*Z N-glucosides, rather than iP N-glucosides, can be metabolized back to *t*Z free base forms [62], showing a novel mechanism for regulating bioactive cytokinin levels, with *t*Z N-glucosides functioning as a readily accessible storage form rather than merely an irreversible inactivation product [62,128,146,147].

O-glucosides, linked to antioxidant and anti-inflammatory properties, and N-glucosides, associated with nitrogen storage and herbivore defence, may offer additional therapeutic benefits [145,148,149]. CK forms such as *t*Z7G and *t*Z9G act as storage reservoirs, releasing active cytokinins under specific conditions, which could be harnessed for sustainable therapeutic applications [150,151]. The higher CK concentrations and diverse metabolite profiles of the red ecotype provide insights into its enhanced bioactivity, offering a foundation for applications in natural medicine, dietary supplements, and pharmaceutical developments [145,150,152,153,154]. These findings underscore the need for further research to explore its full potential in human health and sustainable practices.

The correlation network analysis revealed positive associations among CKs, including *t*Z, cZ, cZOG, cZ9G and *t*Z9G, which exhibited strong positive correlations with alkaloids such as coniferin and hypoxanthine (Figure 7; Table 3 and Table S7). Alkaloids are a large class of plant secondary metabolites reported to possess antimicrobial, analgesic, antimalarial and anticancer activity [155,156,157]. These findings suggest that these CKs may play a significant role in promoting the biosynthesis or accumulation of these compounds, which are frequently associated with the plant’s adaptive mechanisms, including defence and stress tolerance. For example, CKs have been shown to influence the production of tropane alkaloids in the Solanaceae family [158]. The biosynthesis of tropane alkaloids, including tropine, involves complex pathways that can be regulated by plant hormones such as CKs [159]. CKs have been reported to affect alkaloid accumulation in certain plant species, which are crucial for the plant’s defence mechanisms against herbivores and pathogens [160,161]. In another example, *Catharanthus roseus*, Amaryllidaceae species (*Rhodophiala pratensis*, *R. splendens*, *R. advena*, and *Rhodolirium speciosum*) a prominent source of alkaloids, had alkaloid production enhanced through benzylaminopurine (BA) application, potentially through modulating specific biosynthetic pathways [162]. This observation indicates that CKs may function as signaling molecules, promoting metabolic flux toward alkaloid biosynthesis [39,163].

Conversely, CKs such as *c*ZR, *t*ZR, and iP showed significant negative correlations with tropine, trigonelline and pipecolinic acid (Figure 7; Table 3 and Table S7). This trend suggests that these CKs might suppress dopamine biosynthesis or redirect metabolic resources toward other pathways. Such regulatory trade-offs emphasize the balancing act between different secondary metabolite pathways, reflecting the plant’s dynamic response to environmental or physiological factors [157].

Correlations between CKs and secondary metabolites particularly flavonoids and anthocyanins, have been investigated [164,165]. Among the key findings, cZ, *t*Z9G and cZ9G demonstrated a strong positive association with chalconaringenin, naringenin and leucocyanidin (Figure 7), suggesting its role in promoting flavonoid precursor biosynthesis that are essential for plant defence mechanisms and pigmentation [166]. Similarly, *t*ZR exhibited a positive correlation with delphinidin glucosides and cyanidin derivatives, emphasizing its role in enhancing anthocyanin production, crucial for antioxidant activity and stress tolerance [149,151,167,168,169]. Chalcones, including chalconaringenin, are pivotal intermediates in the flavonoid biosynthetic pathway, leading to the production of various flavonoids and anthocyanins. The promotion of chalconaringenin biosynthesis by *c*Z9G indicates a potential mechanism through which CKs can modulate secondary metabolite profiles in plants, enhancing their ability to cope with environmental stresses and attract pollinators through pigmentation [170,171].

A prime example of CK’s influence on flavonoids was reported in a carob study where a combination of the synthetic cytokinin 6-benzyladenine (i.e., BA) and UV-C irradiation resulted in increased soluble flavonoid, flavonol, and hydroxycinnamic acid levels [172]. This metabolite enhancement is often moderated through upregulating flavonoid biosynthetic pathway genes [172], and their critical role linked to redox states in cells, particularly glutaredoxin genes, which suggested a protective function against oxidative stress [67,68]. BA was also reported to boost secondary metabolite biosynthesis and accumulation in *Santalum album* heartwood [173,174], especially in significantly enhancing essential oil, flavonoids, and phenolics production, and upregulated genes involved in terpenoid and flavonoid biosynthesis, showcasing its regulatory effect on metabolic pathways. Additionally, BA synergized with auxins, gibberellins, and jasmonic acid to promote essential oil biosynthesis. Transcriptomic and metabolomic analyses revealed BA activating a complex regulatory network, which induced heartwood formation and improved metabolite production [47,175,176]. These studies show that CKs have an integral role in increasing flavonoid content in plants, supporting our results of increased correlation of phytohormones with flavonoids.

Previously, the effects of varying nitrogen (N) conditions on the accumulation of flavonoids and phytohormones in *Camellia sinensis* tea plants were investigated [177]. Although the results indicated complex correlation patterns, certain flavonoid compounds (taxifolin, myricetin, and apigenin) were positively correlated with CKs, such as iP, *c*Z and *t*ZR [177]. Furthermore, the interaction between CKs and flavonoid biosynthesis has been linked to improved antioxidant activity in plants, which is essential for mitigating oxidative stress. This highlights the broader implications of CKs in enhancing plant resilience and adaptability through the regulation of secondary metabolites [170,177].

Several notable correlations between phenolic compounds and CKs were identified. Among the most significant relationships, vanillic acid (Figure 7; Table 3, Tables S4, S5 and S8) demonstrated strong positive correlations with multiple CKs, appearing as orange-brown squares in the heatmap (Figure 7). 5-O-(4-Coumaroyl)-D-quinate also shows distinct positive correlations across several CKs (Figure 7). Tetrahydrofolate exhibited a mixed pattern of correlations, with some strong positive associations indicated by orange colours and some negative correlations shown in turquoise. Sinapyl alcohol and sinapaldehyde displayed moderate to strong positive correlations with specific CKs, while *p*-coumaroyl quinic acid showed a more varied correlation pattern across different CK compounds (Figure 7).

The correlations between phenolic compounds and CKs highlight significant regulatory interactions in plant secondary metabolism. Among these, vanillic acid showed strong positive correlations with multiple CKs [178] such as *t*Z, *t*ZR and *t*ZROG (Figure 7; Table 3 and Table S8). This suggests that CKs may play a key role in modulating vanillic acid biosynthesis, which is a phenolic compound with well-known antioxidant properties [179,180]. Such interactions could imply a broader regulatory network where CKs influence phenolic pathways to enhance plant stress responses or metabolic activity [181,182,183]. Additionally, 5-O-(4-coumaroyl)-D-quinate, a precursor in the biosynthesis of lignin and other phenylpropanoids, also demonstrates consistent positive correlations with several CKs (Figure 7). This points to CKs as potential regulators of lignin biosynthesis and structural phenolics critical for plant integrity and defence mechanisms. Tetrahydrofolate exhibited a more mixed correlation profile, with both strong positive (orange) and negative (turquoise) associations, indicating a nuanced role of CKs in its metabolic pathways. Compounds such as sinapyl alcohol and sinapaldehyde showed moderate to strong positive correlations with specific CKs, suggesting their biosynthesis might be selectively regulated by particular CK derivatives. In contrast, *p*-coumaroyl quinic acid displayed a varied pattern of correlations across different CKs, hinting at a complex interplay that may involve context-specific regulation or competing metabolic priorities. These findings emphasize the multifaceted role of CKs in fine-tuning the production of phenolics, with implications for plant stress tolerance, structural adaptation, and metabolic optimization.

### 4.2. Metabolome Diversity in D. dentatus’ Ecotypes Hints to Therapeutic Potential

Metabolic differences were revealed between *D. dentatus*’ red and white ecotypes, particularly in secondary metabolite biosynthesis with potential health benefits (Figure 8). The red ecotype demonstrated 20 metabolic pathways, while the white ecotype showed 16 pathways, highlighting the plant’s complex biochemical capabilities and ecological adaptations. Key pathways such as flavonoid, flavone, flavonols, anthocyanin, and monoterpenoid biosynthesis were found to be highly significant. The red ecotype demonstrated higher activity in flavone, flavonols and anthocyanin biosynthesis (Figure 8; *p* = 0.003–0.060), compounds known for their antioxidant and anti-inflammatory properties, while the white ecotype was more associated with monoterpenoid and flavonoid biosynthesis. Flavonoids, such as quercetin, have been extensively studied for their ability to combat oxidative stress, reduce inflammation, exhibit anticancer properties, and prevent cell ageing [84,184,185]. Anthocyanins, pigments responsible for the red and purple hues in plants, were particularly abundant in the red ecotype and are linked to anti-inflammatory, anticancer, and neuroprotective effects [185,186,187]. Meanwhile, monoterpenoids in the white ecotype, recognized for their antimicrobial, anti-inflammatory, and analgesic properties, further highlight the plant’s therapeutic potential [188]. The categorization of metabolites into nitrogen-containing compounds (e.g., alkaloids) and nitrogen-deficient compounds (e.g., terpenoids and phenolics) underscores the plant’s broad chemodiversity. Alkaloids have been used for centuries in medicine for their diverse pharmacological activities, including analgesic, antimalarial, and anticancer properties, while phenolics have been associated with anti-inflammatory, antidiabetic, and cardioprotective benefits [189].

Additionally, phytohormone profiling showed higher concentrations of active CKs, such as *t*Z and cZ, in the red ecotype, alongside glucoside forms such as *t*Z7G, which may serve as reserves for activation under specific conditions. The untargeted metabolomics analysis previously published further revealed a greater number of upregulated metabolites in the red ecotype, with polyphenols accounting for 63.9% of identified compounds in both ecotypes [9]. This abundance of polyphenols, particularly in the red ecotype, underscores the potential therapeutic value of *D. dentatus*, given their well-documented antioxidant and anti-inflammatory properties [84,184]. Together, these metabolic and phytochemistry differences between the ecotypes illustrate their unique adaptations and underscore *D. dentatus* as a promising source of bioactive compounds for medicinal applications.

## 5. Conclusions

This study highlighted, for the first time, phytohormone profiling in *D. dentatus*. In addition, it highlighted the correlation of endogenous phytohormones with select alkaloids, flavonoids and other phenolic compounds. Although a small glimpse of the plant’s therapeutic potential is shown, future research on *D. dentatus* should prioritize the quantification of phenolic compounds in both red and white ecotypes while also considering the ecological and environmental factors that contribute to the metabolic variations between them. By doing so, valuable insights can be gained regarding the adaptive mechanisms of these ecotypes and their potential resilience in the face of climate change. Additionally, by focusing on the biosynthesis pathways of key metabolites such as flavonoids, anthocyanins, and monoterpenoids, we can uncover the regulatory genes and enzymes involved, potentially paving the way for biotechnological and possible therapeutic applications. Further investigations of the bioavailability and therapeutic effectiveness of these metabolites in clinical settings would also substantiate their potential benefit to human health. Furthermore, it is important to delve deeper into the physiological relevance of CK glucosides, specifically *t*Z7G, as reservoirs for active CKs under stressful conditions. Lastly, broadening the scope of metabolomic and genomic analyses to encompass related species would enhance our understanding of both the conserved and distinctive characteristics of secondary metabolites in *D. dentatus*, thereby contributing to our understanding of the evolutionary importance of these compounds.

*D. dentatus*’ cytokinin levels can be influenced by seasonal variations [190], edaphic factors (i.e., soil conditions; [191]), physiological age [192], and abiotic and biotic factors [129,190]. For example, Guyana has a wet and dry season [193], associated with varying rainfall levels and temperature, and soil nutrient levels varying with location, and elevation; all acting as factors that can influence cytokinin levels. Although looking on factors that could influence cytokinin levels in *D. dentatus* was not the goal of this work, we acknowledge that this is a limitation in our study. Although this work serves as a preliminary screening for detecting and quantifying endogenous phytohormone levels, along with reporting metabolites detected with level 2 and 3 annotation levels, and the correlation of phytohormones with key metabolite classes, these environmental factors by which cytokinins and other phytohormone levels can be influenced will be addressed in future endeavours.

Despite targeted hormonomics showing immense cytokinin potential, especially for the red ecotype having 6.2 times the cytokinin levels when compared to the white ecotype, and the presence of polyphenolic compounds, as previously published [9], prioritizing the harvesting of *D. dentatus*’ red ecotype for therapeutic purposes in a sustainable way, especially in preserving biodiversity, can aid in pharmaceutical research. Although this work showed first the first time the presence and quantification of cytokinins in *D. dentatus* and other metabolites, future studies will investigate the targeted bioactive compound extraction of cytokinins and/or other key metabolites for pharmacological testing.

## Figures and Tables

**Table 1:** CKs and conjugates that were scanned for and detected in either or both of *D. dentatus* red and white ecotypes. 10 ng of each listed internal standard was added to each sample.

**Endogenous CKs**	**Abbreviation**	**^2^H-labelled Internal** **Standards**
*Free bases (CKFB)*		
N^6^-isopentenyladenine	iP	^2^H_6_iP
*trans*-Zeatin	*t*Z	^2^H_3_DZ
*cis*-Zeatin	*c*Z	^2^H_3_DZ
Dihydrozeatin	DZ	^2^H_3_DZ
*Ribosides (CKRB)*		
N^6^-isopentyadenosine	iPR	^2^H_6_[9R]iP
*trans*-Zeatin riboside	*t*ZR	^2^H_5_[9R]*t*Z
*cis*-Zeatin riboside	*c*ZR	^2^H_5_[9R]*t*Z
Dihydrozeatin riboside	DZR	^2^H_3_[9R]DZ
*Glucosides (CKGLUC)*		
isopentenyladenine-7-glucoside	iP7G	^2^H_6_iP
isopentenyladenine-9-glucoside	iP9G	^2^H_6_iP
*trans*-Zeatin-O-glucoside riboside	*t*ZROG	^2^H_5_tZROG
*cis*-Zeatin-O-glucoside riboside	*c*ZROG	^2^H_5_tZROG
*trans*-Zeatin-O-glucoside	*t*ZOG	^2^H_5_tZOG
*cis*-Zeatin-O-glucoside	*c*ZOG	^2^H_5_tZOG
Dihydrozeatin-O-glucoside	DZOG	^2^H_7_DZOG
Dihydrozeatin-O-glucoside riboside	DZROG	^2^H_7_DZROG
*trans*-Zeatin-9-glucoside	*t*Z9G	^2^H_5_tZ9G
*trans*-Zeatin-7-glucoside	*t*Z7G	^2^H_5_tZ7G
*cis*-Zeatin-9-glucoside	*c*Z9G	^2^H_5_tZ9G
Dihydrozeatin-9-glucoside	DZ9G	^2^H_3_DZ9G

**Table 2:** CKs and other phytohormones that were scanned for but not detected in either ecotype of *D. dentatus*. Internal standards added to each sample: 10 ng of CKs, IAA and SA; 20 ng of Gas, 60 ng of ABA.

**Endogenous CKs**	**Abbreviation**	**^2^H-labelled Internal** **Standards**
*Methylthiol-CKs (2MeS-CK)*		
2-Methylthio-N^6^-isopentayladenine	2MeSiP	^2^H_6_2MeSiP
2-Methylthio-N^6^-isopentenyladenosine	2MeSiPR	^2^H_6_2MeSiPR
2-Methylthio-zeatin	2MeSZ	^2^H_5_2MeStZ
2-Methylthio-zeatin riboside	2MeSZR	^2^H_5_2MeStZR
*Aromatics*		
Kinetin	KIN	^2^H_7_BA
N^6^-benzyladenine	BA	^2^H_7_BA
N^6^-benzyladenosine	BAR	^2^H_7_[9R]BA
*Acidic Phytohormones*		
Abscisic acid	ABA	[^2^H_4_]ABA
Indole-3-Acetic Acid	IAA	[^2^H_5_]IAA
Salicylic acid	SA	[^2^H_4_]SA
Gibberellin A_1_	GA_1_	[^2^H_4_]GA_1_
Gibberellin A_4_	GA_4_	[^2^H_2_]GA_4_
Gibberellin A_7_	GA_7_	[^2^H_2_]GA_7_
Gibberellin A_9_	GA_9_	[^2^H_2_]GA_9_
Gibberellin A_20_	GA_20_	[^2^H_2_]GA_20_

**Table 3:** Targeted metabolomics analysis of detected cytokinin phytohormones in methanol and chloroform extracts from *D. dentatus*’ red and white ecotypes using LC-MS at MSI level 1 annotation. The reported phytohormone levels represent the averages of biological triplicates with standard errors (pmol*g^−1^/DW ± SE), combining levels found in chloroform and methanol extracts. Meanings of phytohormone abbreviations are listed in Table 1.

**Cytokinin Class**	**Phytohormones**	***D. dentatus*** **(Red Ecotype)**	***D. dentatus*** **(White Ecotype)**
Free bases	iP	0.48 ± 0.12	0.370 ± 0.109
	*c*Z	11.02 ± 2.22	5.82 ± 1.23
	*t*Z	155.48 ± 20.23	84.52 ± 26.03
	DZ	1.56 × 10^−3^ ± 0.00	3.93 × 10^−3^ ± 0.00
Ribosides	*t*ZR	0.69 ± 0.17	0.32 ± 0.03
	cZR	0.07 ± 0.02	0.11 ± 0.05
	DZR	11.85 ± 2.47	6.80 ± 0.74
	iPR	0.06 ± 0.01	0.08 ± 0.01
Glucosides	DZROG	578.59 ± 330.89	494.90 ± 296.07
	DZ9G	39.94 ± 26.30	3.09 × 10^−2^ ± 0.00
	DZOG	16.70 ± 2.84	12.01 ± 0.54
	*c*ZOG	609.86 ± 115.09	430.99 ± 33.41
	*t*ZOG	292.07 ±62.94	182.32 ± 12.78
	*t*Z9G	112.93 ± 76.23	1.02 ± 0.07
	*c*Z9G	115.77 ± 78.08	1.03 ± 0.07
	*t*Z7G	7161.10 ± 2421.62	0.69 ± 0.19
	iP7G	7.84 ± 5.08	99.12 ± 63.61
	iP9G	52.75 ± 33.25	158.42 ± 92.27
Total CK levels	(pmol*g^−1^/DW ± SE)	9135.49 ± 3174.72	1478.53 ± 527.209

## Data Availability

Data not in the Supplementary Materials section was uploaded on Zenodo at https://zenodo.org/records/16412543 (accessed on 24 July 2025) on 24 July 2025.

## Supplementary Materials

The following supporting information can be downloaded at: https://www.mdpi.com/article/10.3390/metabo15080533/s1, Figure S1. A proposed schematic of the cytokinin (CK) biosynthesis pathway in *D. dentatus* (Guyanese Capadulla) woody vine consisting of two activation pathways—De Novo and tRNA degradation. The information presented in the pathway was adapted from [62,194,195,196,197,198] current and previous studies on plants and fungi research. Model for cytokinin biosynthesis in plants. CK biosynthesis starts by transferring an isopentenyl moiety (IptA) catalysis to from dimethylallyl diphosphate (DMAPP) to AMP/ADP/ATP to form free N^6^-isopentenyladenine-type (iP-type) CKs and discadenine (DA) via the De Novo biosynthesis pathway [62]. In Arabidopsis thaliana the isopentenyl moiety is transferred mainly to ATP/ADP. This reaction is catalyzed by the phosphate-isopentenyl transferase (IPTA) enzymes. The initial products iPRMP, iPRDP, and iPRTP are subsequently hydroxylated in the isoprenoid side chain by cytochrome P450 monooxygenases CYP735A (orange box) for the formation of the corresponding tZ nucleotides. IptB and IptC are proposed tRNA isopentenyl transferases which catalyze prenylation of tRNA molecules that can be further modified to form CKs or cis-Zeatin-type (cZ-type) CKs via the tRNA degradation pathway. The ribotides (green box) can be converted to their free bases by two pathways: the two-step activation is catalyzed by 5 -ribonucleotide phosphohydrolase for the formation of ribonucleosides (pink box) and by adenosine nucleosidase for the formation of the active form (green box). So far, only one has been identified, a nucleoside N-ribohydrolase (NRH), which catalyzes the hydrolysis of iPR to iP (orange box). The direct activation pathway is catalyzed by 5′-monophosphate phosphorribohydrolase (orange—active form). The formation of CKs of the *cis*-Zeatin-type begins with the prenylation of adenine 37 on specific (UNN-) tRNAs by tRNA-isopentenyl transferase (tRNA-IPT) (orange box) and subsequent release of CK nucleotides by tRNA degradation [50,87]; Figure S2. The volcano plot from the mass spectrometry data demonstrates the magnitude and significance of the *D. dentatus* red ecotype compared with the control *D. dentatus* white ecotype. The plot visualizes the log_2_ fold change in metabolites expression on the *x*-axis against the −log_10_ *p*-value on the *y*-axis, categorizing metabolites features into downregulated (blue triangles), upregulated (orange triangles), and not significantly changed (grey triangles). The total number of metabolites in each category is indicated in parentheses. The metabolites with higher *p*-values and greater fold changes stand out as the most significantly altered, indicating key differences in metabolite expression between D. dentatus red and white ecotypes; Figure S3. Tentative identification and peak areas of selected flavonoids in *D. dentatus* ecotypes in methanol and chloroform solvent fractions; Figure S4. Tentative identification and peak areas of selected alkaloids in *D. dentatus* ecotypes in methanol and chloroform solvent fractions; Figure S5. Tentative identification and peak areas of selected terpenoids in *D. dentatus* ecotypes in methanol and chloroform solvent fractions; Figure S6. Tentative identification and peak areas of other secondary metabolites in *D. dentatus* ecotypes in methanol and chloroform solvent fractions; Figure S7. Peak areas of selected phytohormones in *D. dentatus ecotypes* in methanol and chloroform solvent fractions; Table S1. Endogenous phytohormones scanned for using the Q-Exactive Orbitrap mass spectrometer, with their classifications and abbreviations alongside isotopically labelled internal standards used for quantification; Table S2. Tentative metabolite identification of selected compounds that are biofingerprints of interest found in both *D. dentatus* red and white ecotypes; Table S3. MSI Level 2 metabolite annotation of compounds in *D. dentatus*’ red and white ecotypes using MS-DIAL v. 5.5. Compounds of interest have an asterisk (*) beside them, and are shaded in yellow; Table S4. MSI Level 2 metabolite annotation of selected compounds in *D. dentatus* using the classical molecular networking (CMN) module of GNPS; Table S5: MSI Level 2 metabolite annotation of selected compounds using the feature based molecular networking (FBMN) module of GNPS; Table S6: Level 3 metabolite annotation of selected compounds in *D. dentatus* using SIRIUS v. 5.6.3 via the mzmine 2.5.3 workflow; Table S7. Pearson’s correlation analysis of endogenous cytokinins with selected alkaloids; Table S8. Pearson’s correlation analysis of endogenous cytokinins with selected flavonoids; Table S9. Pearson’s correlation analysis of endogenous cytokinins with selected phenolic compounds; Table S10. Pathway analysis, encompassing all metabolic pathways from KEGG and HMDB databases with total hits for *D. dentatus*’ red and white ecotypes. Pathways are categorized based on their *p*-values (on the *y*-axis), derived from pathway enrichment analysis, and pathway impact values (on the *x*-axis), obtained from pathway topology analysis. The analysis was performed using the MetaboAnalyst 6.0 platform with the GSEA algorithm, using default settings, and the *Arabidopsis thaliana* pathway library.
