# Supplementary material for: Exploring the Cytokinin Profile of Doliocarpus dentatus (Aubl.) Standl. From Guyana and Its Relationship with Secondary Metabolites: Insights into Potential Therapeutic Benefits
Source: Metabolites. 2025 Aug 6;15(8):533. doi: 10.3390/metabo15080533 (PMC12388203; doi:10.3390/metabo15080533)
Supplement: Supplementary file 1 [file metabolites-15-00533-s001.zip › metabolites-3758089-supplementary.pdf]

Supplemental Information

Exploring the cytokinin profile of *Doliocarpus Dentatus* (Guyanese Capadulla) and its relationship with secondary metabolites: Insights to its potential therapeutic benefits.

Ewart A. Smith[1*], Ainsely Lewis[2,3*], Erin N. Morrison[1,2], Kimberly Molina-Bean[1], Suresh S. Narine[4,5] and R.J. Neil Emery[2]

[1]Environmental and Life Sciences Graduate Program, Trent University, Peterborough, ON K9J 0G2, Canada

[2]Department of Biology, Trent University, Peterborough, ON K9J 0G2, Canada

[3]Department of Chemical and Physical Sciences, University of Toronto Mississauga, Mississauga, ON L5L 1C6, Canada

[4]Trent Centre for Biomaterials Research, Trent University, Peterborough, ON K9J 0G2, Canada

[5]Departments of Physics & Astronomy and Chemistry, Trent University, Peterborough, ON K9J 0G2, Canada

*Corresponding Authors:

Ewart A. Smith; Email: ewartsmith@trentu.ca

Ainsely Lewis; Email: ainselylewis@alumni.trentu.ca; ainsely.lewis@utoronto.ca

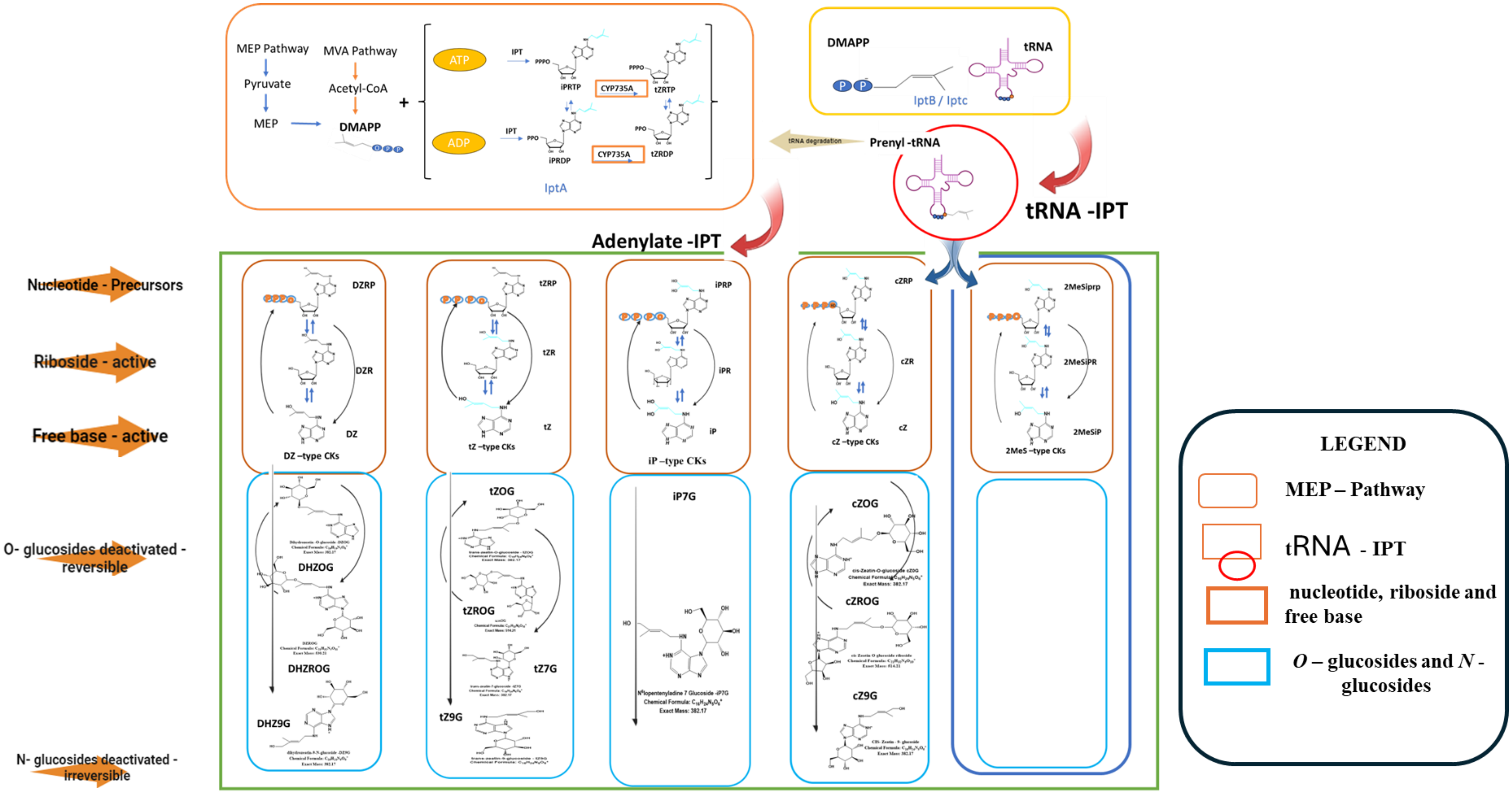

Figure S1. A proposed schematic of the cytokinin (CK) biosynthesis pathway in *D. dentatus* (Guyanese Capadulla) woody vine consisting of two activation pathways—de novo and tRNA degradation. The information presented in the pathway was adapted from [61,187-191] current and previous studies on plants and fungi research. Model for cytokinin biosynthesis in plants. CK biosynthesis starts by transferring an isopentenyl moiety (IptA) catalysis to from dimethylallyl diphosphate (DMAPP) to AMP/ ADP/ATP to form free N6-isopentenyladenine-type (iP-type) CKs and discadenine (DA) via the de novo biosynthesis pathway [61]. In Arabidopsis thaliana the isopentenyl moiety is transferred mainly to ATP/ ADP. This reaction is catalyzed by the phos-phate-isopentenyl transferase (IPTA) enzymes. The initial products iPRMP, iPRDP, and iPRTP are subsequently hydroxylated in the isoprenoid side chain by cytochrome P450 monooxygenases CYP735A (orange box) for the formation of the corresponding tZ nucleotides. IptB and IptC are proposed tRNA isopentenyl transferases which catalyze

prenylation of tRNA molecules that can be further modified to form CKs or *cis*-Zeatin-type (cZ-type) CKs via the tRNA degradation pathway. The ribotides (green box) can be converted to their free bases by two pathways: the two-step activation is catalyzed by 5 -ribonucleotide phosphohydrolase for the formation of ribonucleosides (pink box) and by adenosine nucleosidase for the formation of the active form (green box). So far only one has been identified, a nucleoside N-ribohydrolase (NRH), which catalyzes the hydrolysis of iPR to iP (orange box). The direct activation pathway is catalyzed by 5′-monophosphate phosphor -ribohydrolase (orange – active form). The formation of CKs of the *cis*-Zeatin-type begins with the prenylation of adenine 37 on specific (UNN-) tRNAs by tRNA-isopentenyl transferase (tRNA-IPT) (orange box) and subsequent release of CK nucleotides by tRNA degradation [49,86].

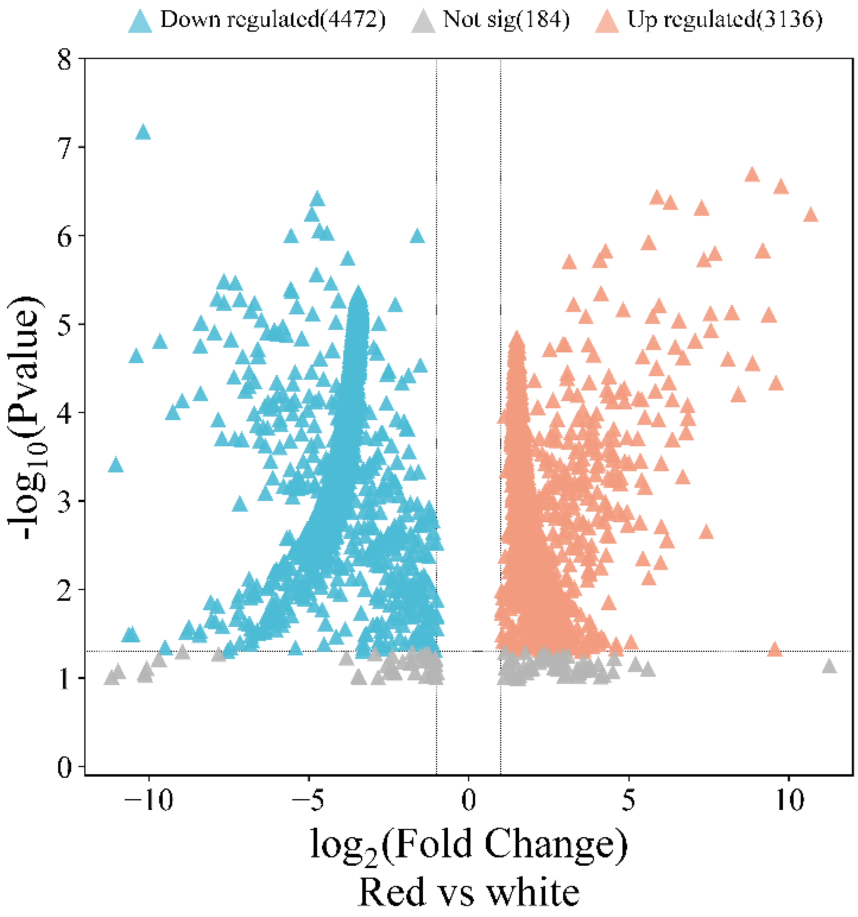

Figure S2. The volcano plot from the mass spectrometry data demonstrates the magnitude and significance of the *D. dentatus* red ecotype compared with the control *D. dentatus* white ecotype. The plot visualizes the $\log_2$ fold change in metabolites expression on the x-axis against the $-\log_{10}$ p-value on the y-axis, categorizing metabolites features into downregulated (blue triangles), upregulated (orange triangles), and not significantly changed (grey triangles). The total number of metabolites in each category is indicated in parentheses. The metabolites with higher p-values and greater fold changes stand out as the most significantly altered, indicating key differences in metabolite expression between D. dentatus red and white ecotypes.

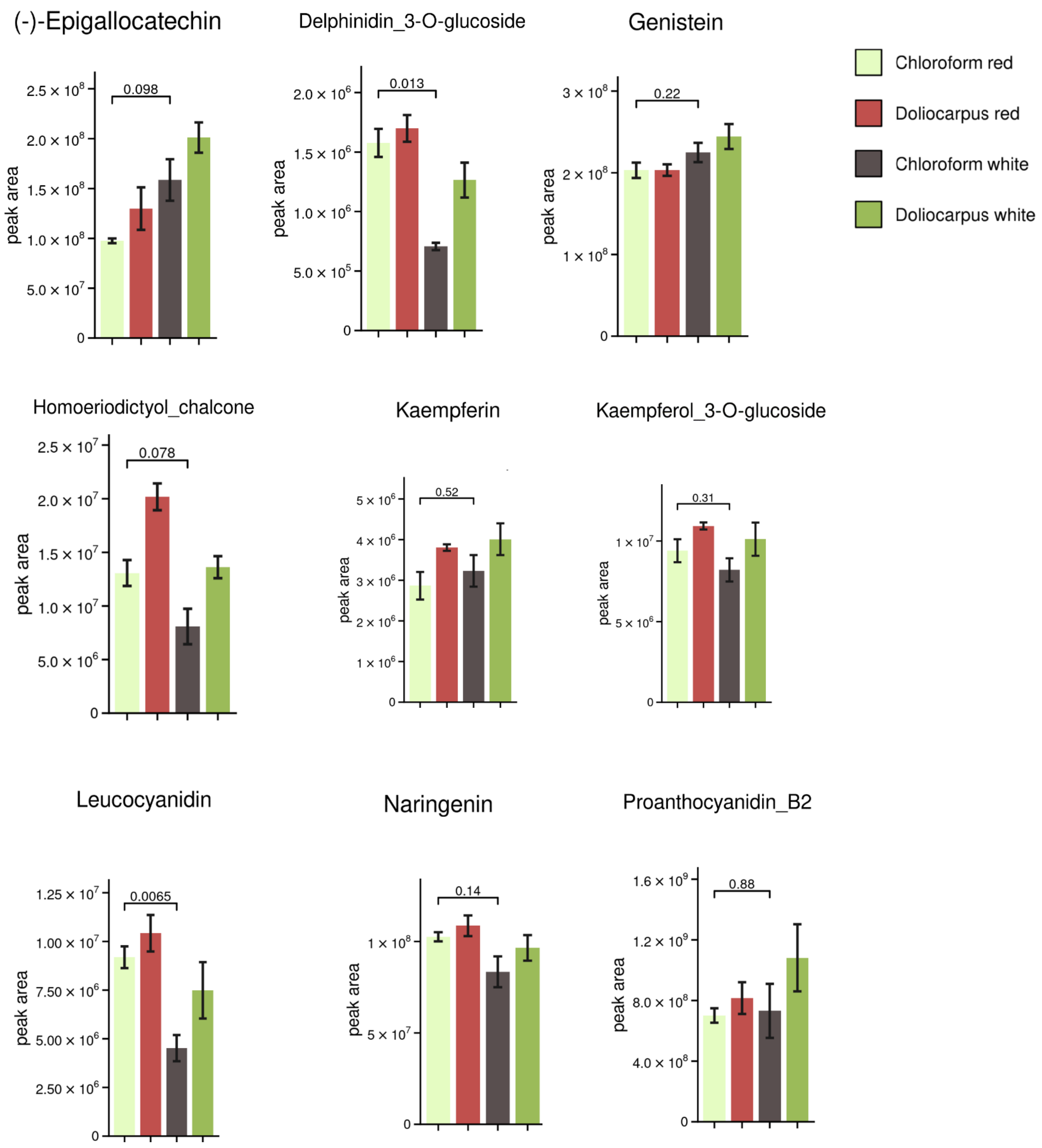

Figure S3. Tentative identification and peak areas of selected flavonoids in *D. dentatus* ecotypes in methanol and chloroform solvent fractions.

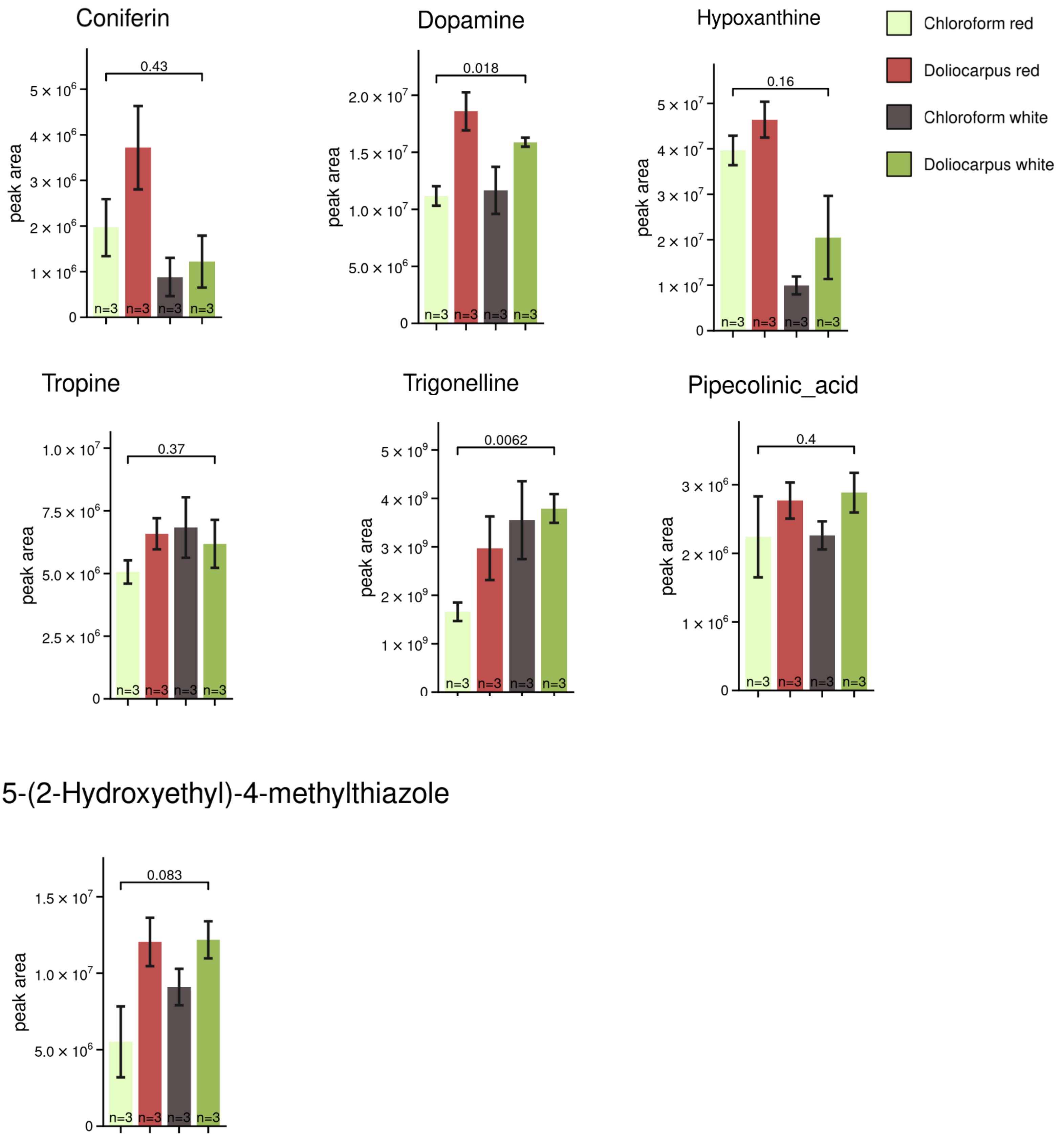

Figure S4. Tentative identification and peak areas of selected alkaloids *in D. dentatus* ecotypes in methanol and chloroform solvent fractions.

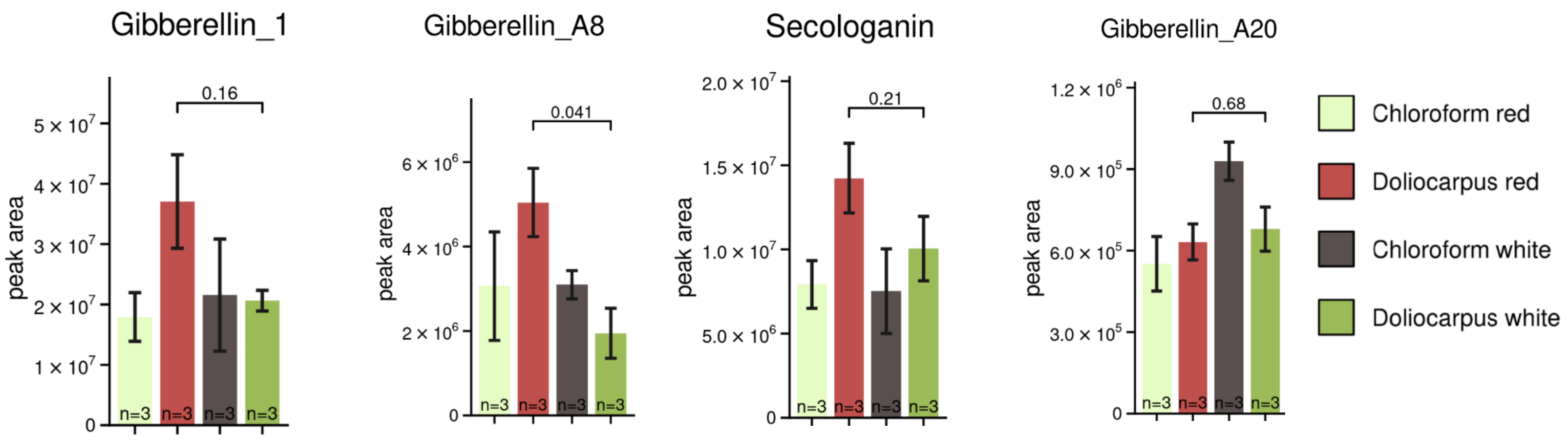

Figure S5. Tentative identification and peak areas of selected terpenoids in *D. dentatus* ecotypes in methanol and chloroform solvent fractions.

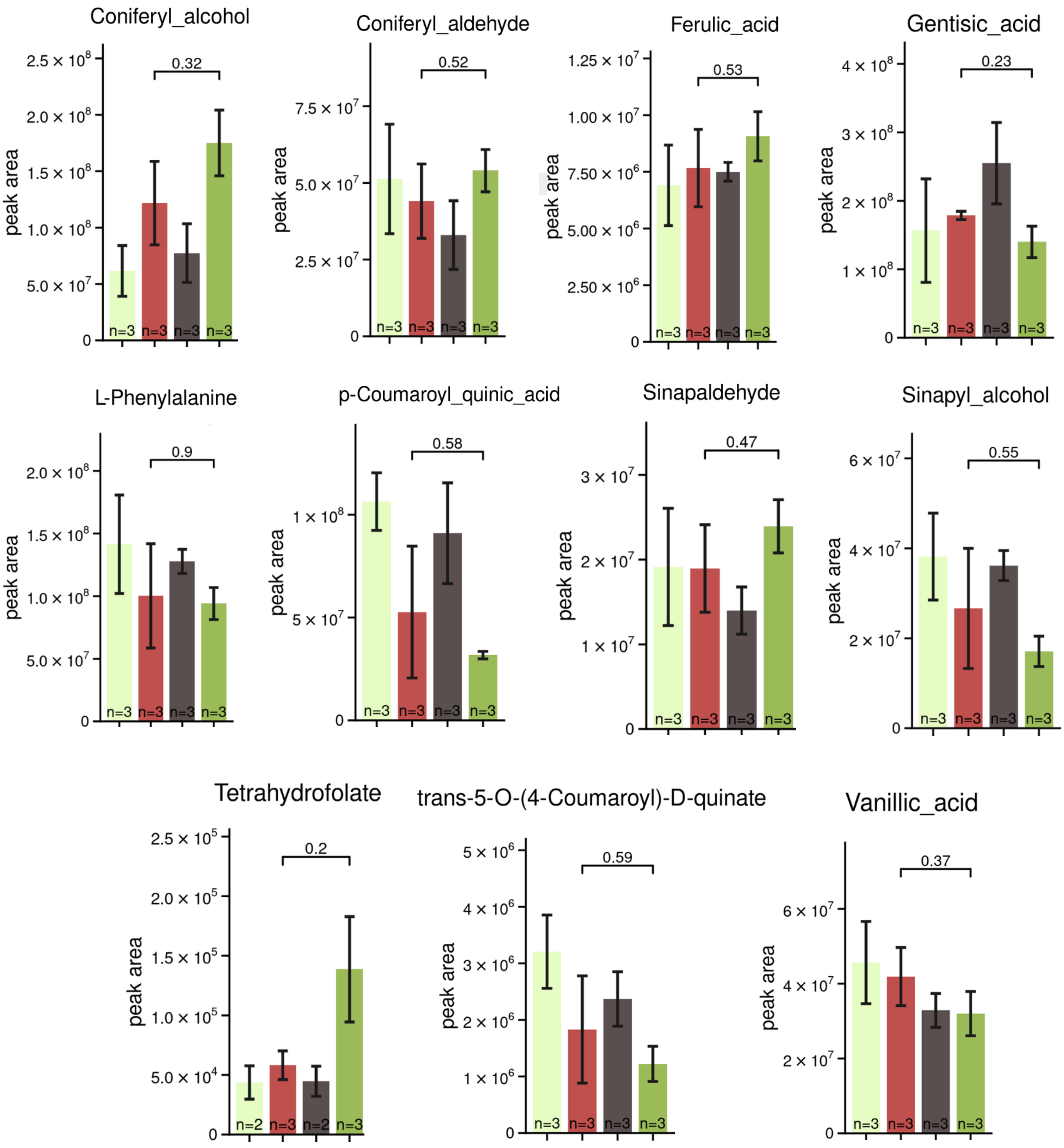

Figure S6. Tentative identification and peak areas of other secondary metabolites in *D. dentatus* ecotypes in methanol and chloroform solvent fractions.

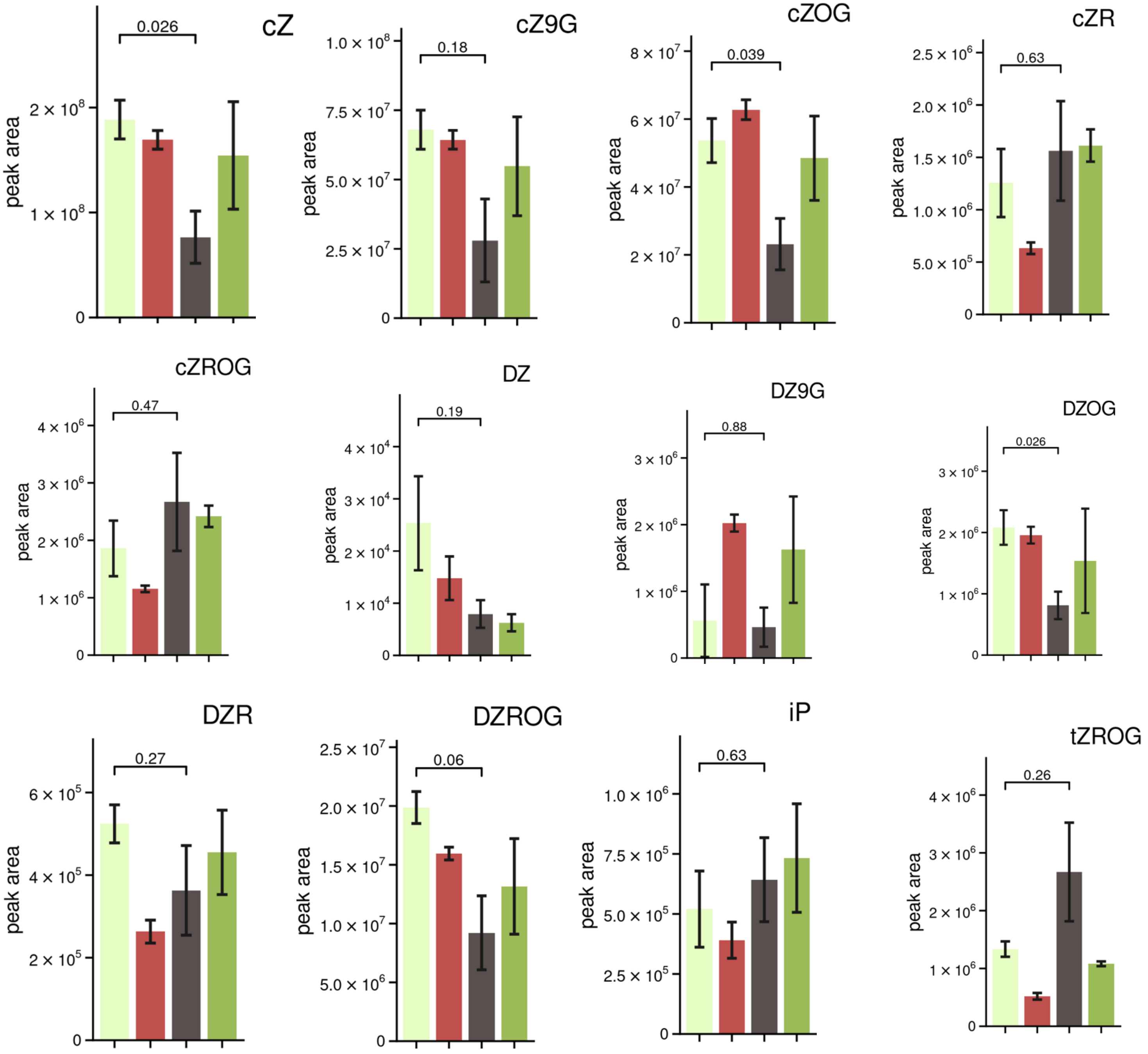

Figure S7. Peak areas of selected phytohormones in *D. dentatus ecotypes* in methanol and chloroform solvent fractions.

Table S1. Endogenous phytohormones scanned for using the Q-Exactive Orbitrap mass spectrometer, with their classifications and abbreviations alongside isotopically labelled internal standards used for their quantification.

Endogenous CK phytohormones	Abbreviation	^{2}H-labelled Internal Standards
**Free bases (CKFB)**		
N^6-isopentenyladenine	iP	$^2H_6$iP
trans-zeatin	*t*Z	$^2H_3$DZ
cis-zeatin	*c*Z	$^2H_3$DZ
Dihydrozeatin	DZ	$^2H_3$DZ
**Ribosides (CKRB)**		
N^6-isopentyadenosine	iPR	$^2H_6$[9R]iP
trans-zeatin riboside	*t*ZR	$^2H_5$[9R]*t*Z
cis-zeatin riboside	*c*ZR	$^2H_5$[9R]*t*Z
Dihydrozeatin riboside	DZR	$^2H_3$[9R]DZ
**Nucleotides (CKNT)**		
N^6-isopentyadenosine-5'-monophosphate	iPRP	$^2H_6$[9RMP]iP
trans-zeatin riboside-5'-monophosphate	*t*ZRP	$^2H_6$[9RMP]DZ
cis-zeatin riboside -5'-monophosphate	*c*ZRP	$^2H_6$[9RMP]DZ
Dihydrozeatin riboside-5'-monophosphate	DZRP	$^2H_6$[9RMP]DZ
**Glucosides (CKGLUC)**		
isopentenyladenine-7-glucoside	iP7G	$^2H_6$iP
isopentenyladenine-9-glucoside	iP9G	$^2H_6$iP
trans-zeatin-O-glucoside	*t*ZOG	$^2H_5$tZOG
cis-zeatin-O-glucoside	*c*ZOG	$^2H_5$tZOG
Dihydrozeatin-O-glucoside	DZOG	$^2H_7$DZOG
trans-zeatin-O-glucoside riboside	*t*ZROG	$^2H_5$tZROG
cis-zeatin-O-glucoside riboside	*c*ZROG	$^2H_5$tZROG
Dihydrozeatin-O-glucoside riboside	DZROG	$^2H_7$DZROG
trans-zeatin-9-glucoside	*t*Z9G	$^2H_5$tZ9G
cis-zeatin-9-glucoside	*c*Z9G	$^2H_5$tZ9G
Dihydrozeatin-9-glucoside	DZ9G	$^2H_3$DZ9G
**Methylthiols (2MeS-CK)**		
2-Methylthio- N^6-isopentayladenine	2MeSiP	$^2H_6$2MeSiP
2-Methylthio-N^6-isopentenyladenosine	2MeSiPR	$^2H_6$2MeSiPR
2-Methylthio-zeatin	2MeSZ	$^2H_5$2MeStZ
2-Methylthio-zeatin riboside	2MeSZR	$^2H_5$2MeStZR
**Aromatics**		
Kinetin	KIN	$^2H_7$BA
N^6-benzyladenine	BA	$^2H_7$BA
N^6-benzyladenosine	BAR	$^2H_7$[9R]BA
**Acidic Phytohormones**		
Abscisic acid	ABA	[$^2H_4$] ABA
Indole-3-Acetic Acid	IAA	[$^2H_5$] IAA
Jasmonic acid	JA	[$^2H_6$] JA (not added)
Salicylic acid	SA	[$^2H_4$]SA
Gibberellin A_1	GA_1	[$^2H_4$]GA_1
Gibberellin A_4	GA_4	[$^2H_2$]GA_4
Gibberellin A_7	GA_7	[$^2H_2$]GA_7
Gibberellin A_9	GA_9	[$^2H_2$]GA_9

Gibberellin A_{20}	GA_{20}	$[^2H_2]GA_{20}$

Table S2. Tentative metabolite identification of selected compounds that are biofingerprints of interest found in both *D. dentatus* red and white ecotypes.

Tentative Identification	Molecular formula	Adduct	Exact mass	Theoretical mass	Experimental mass	Mass error (ppm)	t_R (min)	Annotation Level
Cyanidin 3-O-glucoside	$C_{21}H_{21}O_{11}$	$[M+H]^+$	449.1084	450.1157	450.1118	8.6	6.0	3
Pelargonin	$C_{27}H_{31}O_{15}$	$[M+H]^+$	595.1663	596.1736	596.1742	1.1	2.2	3
Cyanidin 3-O-(6-O-p-coumaroyl) glucoside	$C_{30}H_{27}O_{13}$	$[M+H]^+$	595.1452	596.1524	596.1521	0.6	5.8	3
Apiforol	$C_{15}H_{14}O_5$	$[M+H]^+$	274.0841	275.0914	275.0913	0.4	6.1	3
(-)-Epigallocatechin	$C_{15}H_{14}O_7$	$[M+H]^+$	306.074	307.0812	307.0799	4.3	2.1	3
Kaempferol 3-O-glucoside	$C_{21}H_{20}O_{11}$	$[M+H]^+$	448.1006	449.1078	449.1080	0.4	6	3
Homoeriodictyol chalcone	$C_{16}H_{14}O_6$	$[M+H]^+$	302.0790	303.0863	303.0890	8.9	5.5	3
Kaempferin	$C_{21}H_{20}O_{10}$	$[M+H]^+$	432.1056	433.1129	433.113	0.2	6	3
Cyanidin 3-O-beta-D-sambubioside	$C_{26}H_{29}O_{15}$	$[M+H]^+$	581.1506	582.1579	582.1551	4.8	5.8	3
Naringenin	$C_{15}H_{12}O_5$	$[M+H]^+$	272.0685	273.0758	273.0756	0.5	6.1	3
4-Coumaroylshikimate	$C_{16}H_{16}O_7$	$[M+H]^+$	320.0896	321.0969	321.0956	3.9	5.7	3
Cyanidin	$C_{15}H_{11}O_6$	$[M+H]^+$	287.0556	288.0628	288.0639	3.7	5.4	3
Naringenin chalcone	$C_{15}H_{12}O_5$	$[M+H]^+$	272.0685	273.0758	273.0749	3.1	6.1	3
Delphinidin 3-O-glucoside	$C_{21}H_{21}O_{12}$	$[M+H]^+$	465.1033	466.1106	466.1051	11.7	6	3
Delphinidin 3-O-beta-D-sambubioside	$C_{26}H_{29}O_{16}$	$[M+H]^+$	597.1456	598.1528	598.1571	-7.1	5.8	3
Dopamine	$C_8H_{11}NO_2$	$[M+H]^+$	153.079	154.0863	154.0856	4.2	2.3	3
Gibberellin A_1	$C_{19}H_{24}O_6$	$[M+H]^+$	348.1573	349.1646	349.1672	7.5	2.1	3
beta-Caryophyllene	$C_{15}H_{24}$	$[M+H]^+$	204.1878	205.1951	205.1945	2.8	2.17	3
Kaurenol	$C_{20}H_{32}O$	$[M+H]^+$	288.2453	289.2526	289.2554	9.7	7	3

(-)-Menthone	$C_{10}H_{18}O$	$[M+H]^+$	154.1358	155.1430	155.1424	4.1	5.8	3
Gibberellin A_{20}	$C_{19}H_{24}O_5$	$[M-H]^-$	332.1624	333.1697	333.1724	8.2	6.4	3
Tetrahydrofolate	$C_{19}H_{23}N_7O_6$	$[M+H]^+$	445.171	446.1783	446.1796	3.0	5.8	3
L-Phenylalanine	$C_9H_{11}NO_2$	$[M+H]^+$	165.1891	166.0863	166.0857	3.3	2.0	3
Coniferyl alcohol	$C_{10}H_{12}O_3$	$[M+H]^+$	180.0786	181.0859	181.0853	3.4	2.1	3
Caffeic acid	$C_9H_8O_4$	$[M+H]^+$	180.0423	181.0495	181.0489	3.6	1.6	3
Ferulic acid	$C_{10}H_{10}O_4$	$[M+H]^+$	194.0579	195.0652	195.0646	3.0	2.1	3
Sinapaldehyde	$C_{11}H_{12}O_4$	$[M+H]^+$	208.0736	209.0808	209.0802	3.0	5.8	3
Cinnamaldehyde	C_9H_8O	$[M+H]^+$	132.0575	133.0648	133.0649	0.8	5.8	3
Coniferyl aldehyde	$C_{10}H_{10}O_3$	$[M+H]^+$	178.063	179.0703	179.0697	3.2	7.2	3
Delphinidin	$C_{15}H_{11}O_7$	M-H	303.0505	302.0432	302.0443	3.6	6.2	3
Epigallocatechin	$C_{15}H_{14}O_7$	M-H	306.074	305.0667	305.0673	2.1	5.8	2
Genistein	$C_{15}H_{10}O_5$	$[M+H]^+$	270.0528	271.0601	271.0590	4.1	5.8	3
Catechin 3-O-Beta-D-Glucopyranoside	$C_{21}H_{24}O_{11}$	$[M+H]^+$	452.1319	453.1391	453.1377	3.2	2.1	3
Leukoefdin	$C_{15}H_{14}O_8$	$[M+H]^+$	322.0689	321.0616	321.0621	1.6	5.9	3
Vanillic acid	$C_8H_8O_4$	$[M+H]^+$	168.0423	167.0350	167.0352	1.3	2.2	3
Gallic acid	$C_7H_6O_5$	$[M-H]^-$	170.0215	169.0142	169.0147	2.6	5.6	3
Catechol	$C_6H_6O_2$	$[M-H]^-$	110.0368	109.0295	109.0298	2.7	2.1	3
Gentisic acid	$C_7H_6O_4$	$[M-H]^-$	154.0266	153.0193	153.0195	1.1	5.8	3
Dopamine	$C_8H_{11}NO_2$	$[M+H]^+$	153.079	154.0863	154.0856	4.3	2.3	3
Trigonelline	$C_7H_7NO_2$	$[M+H]^+$	137.0477	138.0550	138.0545	3.3	1.8	3

Table S3. MSI Level 2 metabolite annotation of compounds in *D. dentatus'* red and white ecotypes using MS-DIAL v. 5.5. Compounds of interest have an asterisk (*) beside them, and are shaded in yellow.

Name	Formula	Adduct	Precursor m/z	Fragments (MS/MS)	Ecotype found	Fraction found
Hydroxyproprionic acid	$C_3H_6O_3$	[M-H]$^-$	89.02478	59.0141	Red and White	Methanol
Glyceraldehyde	$C_3H_6O_3$	[M-H]$^-$	89.0248	59.0140; 71.0141	Red and White	Chloroform
L-Alanine	$C_3H_7NO_2$	[M+H]$^+$	90.05501	55.0546; 72.0447; 73.05249	Red and white	Chloroform
N,N-dimethylglycine	$C_4H_9NO_2$	[M-H]$^-$	102.05678	84.0458	Red and White	Chloroform and Methanol
Serine	$C_3H_7NO_3$	[M-H]$^-$	104.03549	72.00935; 74.0249	White	Chloroform and Methanol
Glycerate/ D-(+)-Glyceric acid	$C_3H_6O_4$	[M-H]$^-$	105.01974	59.0141; 75.0090	Red and White	Chloroform and Methanol
Catechol*	$C_6H_6O_2$	[M-H]$^-$	109.02999	81.0351; 91.0195	Red	Methanol
Citraconic acid	$C_5H_6O_4$	[M-H_2O-H]-	111.00909	67.0192	Red and White	Chloroform and Methanol
Isobutyric acid	$C_4H_8O_2$	[M+Na]+	111.04411	93.0337	Red	Methanol
Cytosine	$C_4H_5N_3O$	[M+H]+	112.05022	69.0448; 95.0239	Red and white	Chloroform and methanol
Fumaric acid	$C_4H_4O_4$	[M-H]$^-$	115.00406	71.0142	Red and White	Chloroform and Methanol
L-Proline	$C_5H_9NO_2$	[M+H]+	116.07034	70.0651	Red and white	Chloroform
		[M-H]$^-$	116.07178	99.92593	White	Chloroform
Valine	$C_5H_{11}NO_2$	[M+H]+	118.08596	55.0545; 72.0807	Red and white	Chloroform
Succinic acid	$C_4H_6O_4$	[M-H]$^-$	117.01969	73.0297; 99.00908	Red and White	Methanol
Erythrolactone	$C_4H_6O_4$	[M-H]$^-$	117.01985	73.0298; 99.9263	Red and White	Chloroform and Methanol
		[M-H]$^-$	118.05142	72.0096; 74.0252; 100.9264	Red and White	Methanol
Threonine	$C_4H_9NO_3$	[M+H]+	120.06559	56.0499; 74.0602; 84.0444; 102.0551	Red	Chloroform
Guanidinoacetic acid	$C_3H_7N_3O_2$	[M+H]+	118.06513	77.0387; 95.0493; 105.0449	Red	Chloroform
Niacin	$C_6H_5NO_2$	[M+H]+	124.03906	80.0493; 96.0443	Red	Methanol
Benzene-1,2,4-triol*	$C_6H_6O_3$	[M-H]$^-$	125.02464	69.03471; 81.0348; 97.02975	Red	Chloroform
Pipecolic acid*	$C_6H_{11}NO_2$	[M+H]$^+$	130.0863	56.0498; 84.0809	Red	Chloroform and methanol
Aspartic acid/ L-Aspartate	$C_4H_7NO_4$	[M-H]$^-$	132.03041	71.01402; 88.0406	White	Chloroform and Methanol

Isoleucine	$C_6H_{13}NO_2$	$[M+H]^+$	132.10152	69.06995; 86.0963	White	Methanol
D-(+)-Malic acid	$C_4H_6O_5$	$[M-H]^-$	133.01511	71.0142; 89.0248; 115.0042	Red and White	Methanol
Adenine	$C_5H_5N_5$	$[M-H]^-$	134.04779	92.02625; 107.0370	Red	Methanol
Threonic acid	$C_4H_8O_5$	$[M-H]^-$	135.03018	59.01394; 75.0088; 89.02452	White	Chloroform and Methanol
p-Hydroxybenzoic acid	$C_7H_6O_3$	$[M-H]^-$	137.02431	92.0478; 93.03484	White	Methanol
Salicylic acid*	$C_7H_6O_3$	$[M-H]^-$	137.02484	93.0348	Red	Chloroform
O-acetyl-L-Serine	$C_5H_9NO_4$	$[M-H]^-$	146.04582	74.0249; 84.0456; 116.0352	White	Chloroform
		$[M-H]^-$	146.0468	102.0568; 128.03627	Red and White	Chloroform and Methanol
L-Glutamic acid	$C_5H_9NO_4$	$[M+H]^+$	148.06039	84.0444; 102.0550; 130.0500	White	Chloroform and methanol
3-hydroxy-glutaric acid	$C_5H_8O_5$	$[M-H]^-$	147.03001	85.0297; 129.0196	White	Methanol
Glutamine	$C_5H_{10}N_2O_3$	$[M+H]^+$	147.07643	84.0445; 101.0711; 130.0501	White	Chloroform
(R,R)-Tartaric acid	$C_4H_6O_6$	$[M-H]^-$	149.00983	59.0141; 72.99342; 87.0092	White	Methanol
Atranol*	$C_8H_8O_3$	$[M-H]^-$	151.04073	123.04575	Red and White	Chloroform and Methanol
3,4-Dihydroxybenzoic acid	$C_7H_6O_4$	$[M-H]^-$	153.01955	109.0298	Red	Chloroform
Pyrocatechuic acid*	$C_7H_6O_4$	$[M-H]^-$	153.01958	109.0298	White	Methanol
Nonanoic acid/Pelargonic acid	$C_9H_{18}O_2$	$[M-H]^-$	157.12427	113.0977	Red	Methanol
Phenylalanine	$C_9H_{11}NO_2$	$[M+H]^+$	166.08589	103.0541; 120.0806; 131.0490	Red and White	Methanol
Vanillic acid*	$C_8H_8O_4$	$[M-H]^-$	167.03557	108.02234; 123.0458; 152.01224	Red and White	Chloroform and Methanol
Gallic acid*	$C_7H_6O_5$	$[M-H]^-$	169.015	97.02978; 125.0248	Red	Chloroform
N-alpha-Acetyl-L-ornithine	$C_7H_{14}N_2O_3$	$[M-H]^-$	173.09335	129.1035; 131.0828	White	Chloroform
L-Ascorbic acid	$C_6H_8O_6$	$[M+H]^+$	177.0396	95.0130; 113.0235; 141.0186	Red	Methanol
D-sorbosonic acid	$C_6H_{10}O_7$	$[M-H]^-$	193.03564	59.0139; 103.0038	White	Chloroform
Citrulline	$C_6H_{13}N_3O_3$	$[M+Na]^+$	198.08511	138.0528; 153.0636; 181.05861	Red and White	Chloroform

		$[2M-H]^-$	349.18512	52.7649; 131.0828; 174.08861	White	Chloroform and Methanol
Mucic acid	$C_6H_{10}O_8$	$[M-H]^-$	209.03113	85.0298; 133.0148	Red and White	Chloroform
myo-Inositol	$C_6H_{12}O_6$	$[M+CH_3COO]^-$	239.07796	59.0140; 117.0195; 179.0565	White	Methanol
Glycerophosphocholine	$C_8H_{20}NO_6P$	$[M+H]^+$	258.1077	60.0809; 104.1068; 124.99967; 184.0731	Red	Methanol
Galactose 1-phosphate	$C_6H_{13}O_9P$	$[M-H]^-$	259.02264	78.9592; 96.96973; 138.9803	White	Chloroform
alpha-D-(+)-mannose-1-phosphate	$C_6H_{13}O_9P$	$[M-H]^-$	259.02356	78.9593; 96.96996	Red and White	Chloroform and Methanol
Adenosine	$C_{10}H_{13}N_5O_4$	$[M+H]^+$	268.10233	136.06157	White	Chloroform
(+)-Catechin*	$C_{15}H_{14}O_6$	$[M+H]^+$	291.08621	123.04411; 139.0391; 165.0548	Red and White	Chloroform and methanol
Uridine-5-monophosphate	$C_9H_{13}N_2O_9P$	$[M-H_2O-H]^-$	305.02454	59.0141; 141.0174	Red	Chloroform
Nicotinamide mononucleotide	$C_{11}H_{15}N_2O_8P$	$[M+H]^+$	335.06335	97.02843; 123.0552	Red	Methanol
D-(+)-Trehalose	$C_{12}H_{22}O_{11}$	$[M-H]^-$	341.10986	59.01392; 71.01405; 89.0247; 101.02461; 119.03535	White	Methanol
Adenosine 5'-monophosphate	$C_{10}H_{14}N_5O_7P$	$[M-H]^-$	346.0574	78.9592; 96.9698; 134.04744	White	Chloroform
		$[M+H]^+$	348.07034	97.0286; 136.0620	Red and White	Chloroform
Melibiose	$C_{12}H_{22}O_{11}$	$[M+Na]^+$	365.10452	203.0524; 305.0838	Red and White	Chloroform and methanol
Sucrose	$C_{12}H_{22}O_{11}$	$[M+Na]^+$	365.10544	185.04231; 203.0530	White	Chloroform
Raffinose	$C_{18}H_{32}O_{16}$	$[M-H]^-$	503.16336	59.01385; 89.9245; 233.0676	White	Chloroform
		$[M+Na]^+$	527.15918	185.0424; 203.0530; 365.1061	White	Chloroform
Melezitose	C18H32O16	$[M+Na]^+$	527.15845	185.0424; 203.0530; 365.1060	Red	Chloroform

Table S4. MSI Level 2 metabolite annotation of selected compounds in *D. dentatus* using the classical molecular networking (CMN) module of GNPS.

Compound	Molecular Formula	Adduct	Precursor Mass	Fragments (MS/MS)	Fraction Found	Cosine Score
Pyrogallol	$C_6H_6O_3$	$[M+H]_+$	127.04	81.03; 109.0	Chloroform and Methanol	0.79
Salicylic acid	$C_7H_6O_3$	$[M+H]_+$	139.04	65.04; 93.03; 111.0	Chloroform and Methanol	0.84
Homovanillyl alcohol	$C_9H_{12}O_3$	$[M+H-H_2O]^+$	151.07	91.05; 95.05; 119.05	Chloroform and Methanol	0.81
3,4 Dihydroxymandelate	$C_8H_8O_5$	$[M+H-H_2O]^+$	167.03	111.04; 121.04; 139.04	Chloroform and Methanol	0.60
Vanillic acid	$C_8H_8O_4$	$[M+H]^+$	169.05	65.04; 93.03; 111.04; 125.06	Chloroform and Methanol	0.62
Gallic acid	$C_7H_6O_5$	$[M+H]^+$	171.03	81.03; 109.03; 125.02; 127.0	Chloroform and Methanol	0.85
Esculetin	$C_9H_6O_4$	$[M-H]^-$	177.02	105.04; 133.0	Methanol	0.73
Coniferyl aldehyde	$C_{10}H_{10}O_3$	$[M+H]^+$	179.07	55.02; 119.05; 123.04; 133.08; 147.0	Chloroform and Methanol	0.72
5-Hydroxyisovanillic acid	$C_8H_8O_5$	$[M+H]^+$	185.04	81.04; 109.03; 126.0; 141.06; 153.06	Chloroform	0.65
Genipin	$C_{11}H_{14}O_5$	$[M+H]^+$	209.08	93.07; 121.07; 149.06; 177.06	Chloroform and Methanol	0.67
alpha-Santonin	$C_{15}H_{18}O_3$	$[M+H]^+$	247.13	70.06; 112.0; 135.12	Chloroform	0.72
(+/-)-Catechin/Epicatechin	$C_{15}H_{14}O_6$	$[M+H]^+$	289.07	123.0; 135.04; 187.04	Chloroform and Methanol	0.98
Gallocatechin	$C_{15}H_{14}O_7$	$[M-H]^-$	305.07	109.03; 125.0; 139.0; 165.02; 219.07	Methanol	0.64
Epigallocatechin	$C_{15}H_{14}O_7$	$[M+H]^+$	307.08	139.0; 151.04; 289.07	Chloroform	0.81
Hydroxytyrosol glucuronide	$C_{14}H_{18}O_9$	$[M-H]^-$	329.09	59.01; 71.01; 89.03; 123.0; 153.04	Chloroform and Methanol	0.70
Camptothecin	$C_{20}H_{16}N_2O_4$	$[M+Na]^+$	371.1	73.05; 91.05; 267.00; 285.01	Chloroform and Methanol	0.92
Osmanthuside H	$C_{19}H_{28}O_{11}$	$[M-H]^-$	431.15	59.01; 71.01; 89.03; 101.03	Chloroform	0.69
Flavanomarein	$C_{21}H_{22}O_{11}$	$[M-H]^-$	451.13	179.03; 271.06; 289.0	Chloroform	0.67
Eriodictyol-7-O-glucoside	$C_{21}H_{22}O_{11}$	$[M-H]^-$	451.13	179.03; 205.05; 289.0	Methanol	0.61

Procyanidin B1	$C_{30}H_{26}O_{12}$	$[M-H]^-$	577.13	125.0; 127.03; 161.03; 245.08; 289.07; 407.08	Chloroform and Methanol	0.86
Procyanidin B2	$C_{30}H_{26}O_{12}$	$[M+H]^+$	579.15	123.04; 127.0; 163.04; 247.06; 287.06; 409.09	Chloroform and Methanol	0.94

Table S5. MSI Level 2 metabolite annotation of selected compounds using the feature based molecular networking (FBMN) module of GNPS.

**Compound**	**Molecular formula**	**Adduct**	**Precursor m/z**	**Fragments (MS/MS)**	**Cosine Score**
Salicylic acid	$C_7H_6O_3$	$[M+H]^+$	139.04	65.04; 93.03; 111.04	0.95
Homovanillyl alcohol	$C_9H_{12}O_3$	$[M+H-H_2O]^+$	151.07	91.05; 95.05; 119.05	0.65
Genistic Acid	$C_7H_6O_4$	$[M+H]^+$	155.03	65.04; 93.03; 113.96; 131.97	0.87
Gallic Acid	$C_7H_6O_5$	$[M-H]^-$	169.01	81.03; 97.03; 125.02	0.99
Coniferyl aldehyde	$C_{10}H_{10}O_3$	$[M+H]^+$	179.06	55.02; 91.05; 105.07; 119.05; 147.04; 161.06; 179.03	0.73
Quinic acid	$C_7H_{12}O_6$	$[M-H]^-$	191.06	85.03; 87.01; 93.03; 111.01; 127.04	0.71
alpha-Santonin	$C_{15}H_{18}O_3$	$[M+H]^+$	247.13	70.07; 112.08;135.12	0.65
(2R,3S,4S,5R,6S)-2-(hydroxymethyl)-6-(3-hydroxy-5-methylphenoxy)oxane-3,4,5-triol	$C_{13}H_{18}O_7$	$[M-H]^-$	285.1	71.01; 101.02; 113.03; 123.05	0.90
Epicatechin	$C_{15}H_{14}O_6$	$[M-H]^-$	289.09	109.03; 125.03; 137.03; 179.03; 203.07; 245.08	0.96
Epicatechin	$C_{15}H_{14}O_6$	$[M+H]^+$	291.09	119.05; 123.04; 139.04; 147.04; 165.05; 207.06	0.98
Dianthoside	$C_{12}H_{16}O_8$	$[M+H]^+$	289.1	85.03; 97.03; 109.03; 127.0	0.91
Picraquassioside D	$C_{13}H_{18}O_8$	$[M+H]^+$	303.12	85.03; 109.03; 127.04; 141.07	0.86
trans-Zeatin-O-glucoside	$C_{16}H_{23}N_5O_6$	$[M+H]^+$	382.17	136.06; 202.11; 220.12	0.74
(2S,3R,4S,5R)-2-[(2R,3R,4S,5S,6R)-4,5-dihydroxy-6-(hydroxymethyl)-2-(2-phenylethoxy)oxan-3-yl]oxyoxane-3,4,5-triol	$C_{19}H_{28}O_{10}$	$[M-H]^-$	415.15	59.01; 89.02; 101.02; 119.03	0.79
2-methyl-3-[[6-O-[(2R,3R,4R)-tetrahydro-3,4-dihydroxy-4-(hydroxymethyl)-2-furanyl]-beta-D-glucopyranosyl]oxy]-4H-Pyran-4-one	$C_{17}H_{24}O_{12}$	$[M+H]^+$	421.14	127.0; 289.11	0.92

(2R,3R,4S,5S,6R)-2-[[2-(3,4-dihydroxyphenyl)-5,7-dihydroxy-3,4-dihydro-2H-chromen-3-yl]oxy]-6-(hydroxymethyl)oxane-3,4,5-triol	$C_{21}H_{24}O_{11}$	$[M+H]^+$	453.14	85.03; 97.03; 123.04; 139.04; 165.05; 291.09	0.93
Kanokoside A	$C_{21}H_{32}O_{12}$	$[M-H]^-$	475.17	71.01; 89.03; 101.02; 113.03	0.75

Table S6. Level 3 metabolite annotation of selected compounds in *D. dentatus* using SIRIUS v. 5.6.3 via the mzmine 2.5.3 workflow

Compound	Molecular Formula	Adduct	Precursor Mass	Fragments (MS/MS)	Zodiac Score [%]	SIRIUS Score [%]
2-Furoate	$C_5H_4O_3$	$[M+H]^+$	113.0231	57.0337; 67.0178; 69.0337; 95.0128	100.00	91.47
6-Carboxypyrone-2	$C_6H_4O_4$	$[M+H]^+$	141.0183	85.0285; 95.0130; 113.0231	100.00	67.52
2,5-Dihydroxyanisol	$C_7H_8O_3$	$[M+H]^+$	141.0552	95.0495; 123.0442; 126.0332; 127.0391	100.00	87.31
3-imidazol-1-ylpropane-1,2-diol	$C_6H_{10}N_2O_2$	$[M+H]^+$	143.0813	81.0448; 95.0603; 125.0708	100.00	89.74
p-Cresotic acid	$C_8H_8O_3$	$[M+H-H_2O]^+$	153.0546	67.0544; 79.0543; 91.0542; 107.0488;	100.00	78.57
Gallate/Gallic acid	$C_7H_6O_5$	$[M-H]^-$	169.0151	81.0350; 97.0298; 107.0144; 125.0240	100.00	100.00
L-Quinate/Quinic acid	$C_7H_{12}O_6$	$[M-H]^-$	191.0562	85.0294; 87.0088; 111.0091; 127.0596	100.00	99.35
Ferulate/Ferulic Acid	$C_{10}H_{10}O_4$	$[M+H-H_2O]^+$	195.0652	91.0547; 93.0701; 103.0545; 117.0541	99.85	89.01
Pyrogallol A	$C_{12}H_{12}O_6$	$[M+H]^+$	253.0693	69.0339; 97.0289; 109.0289; 127.0389	100.00	49.77
Catechin/Catergen	$C_{15}H_{14}O_6$	$[M+H]^+$	291.0868	123.0436; 139.0394; 147.0441; 165.0538	100.00	100.00
3-(7-Methoxy-4-oxochromen-3-yl)prop-2-ynyl benzoate	$C_{20}H_{14}O_5$	$[M+H]^+$	335.0927	159.0222; 177.0329	82.92	69.64
7-hydroxycoumarin glucuronide	$C_{15}H_{14}O_9$	$[M+H-H_2O]^+$	339.0711	162.9882; 321.0594	40.25	69.63
Isoferulic acid 3-O-|A-D-glucuronide	$C_{16}H_{18}O_{10}$	$[M+H-H_2O]^+$	371.0973	83.0858; 97.1011	14.87	81.47
[(Z)-2-hydroxy-3-(3,4,5-trihydroxybenzoyl)oxyprop-2-enyl] 3,4,5-trihydroxybenzoate	$C_{17}H_{14}O_{11}$	$[M+H-H_2O]^+$	395.0609	57.0703; 377.0489	19.15	69.64
6-[3,4-Dihydroxy-5-(hydroxymethyl)oxolan-2-yl]-5,7-dihydroxy-2-(4-hydroxyphenyl)chromen-4-one	$C_{20}H_{18}O_9$	$[M+H-H_2O]^+$	403.1024	205.0283; 254.0389; 265.0500	100.00	77.14

(2R)-3-Hydroxy-2-[4-(3-hydroxypropyl)-2-methoxyphenoxy]propyl beta-D-glucopyranoside	$C_{19}H_{30}O_{10}$	$[M+H]^+$	419.1922	105.0706; 133.0650; 137.0603; 165.0905	95.93	71.84
Catechin 3-O-beta-D-glycopyranoside	$C_{21}H_{24}O_{11}$	$[M+H]^+$	453.1404	123.0437; 139.0396; 147.0442; 165.0540	99.80	97.66
6-deoxy-Ido(a1-6)Glc(b)-O-galloyl	$C_{19}H_{26}O_{14}$	$[M-H]^-$	477.1274	125.0241;143.0353; 151.0037; 169.0150	93.52	76.67
Kanokoside B	$C_{21}H_{34}O_{12}$	$[M-H]^-$	477.1972	59.0138; 71.0143; 75.0088; 125.0240; 325.1131	99.75	53.62
4,5-Bis[3-(3,4-dihydroxyphenyl)prop-2-enoyloxy]-2,6-dihydroxy-7-methoxy-7-oxohept-2-enoic acid	$C_{26}H_{24}O_{14}$	$[M+H]^+$	561.1219	177.0329; 191.0124; 219.0450; 311.0547	99.90	50.37
Cyanidin-3(6''''-p-coumarylsambibioside)	$C_{35}H_{34}O_{17}$	$[M-H]^-$	725.1732	125.0292; 183.0292; 289.0726	1.11	49.67

Table S7. Pearson's correlation analysis of endogenous cytokinins with selected alkaloids.

Alkaloids	Cytokinins	Correlation value	p-value
**5-(2-Hydroxyethyl)-4-methylthiazole**	cZ	-0.128	0.693
	cZ9G	-0.079	0.818
	cZOG	-0.007	0.982
	cZR	-0.056	0.863
	cZROG	-0.020	0.952
	DZ	-0.501	0.097
	DZ9G	0.713	0.009
	DZOG	-0.163	0.612
	DZR	-0.238	0.456
	DZROG	-0.166	0.607
	iP	0.094	0.771
	iP7G	-0.763	0.006
	iP9G	-0.587	0.298
	iPR	-0.143	0.657
	tZ	-0.668	0.018
	tZ7G	-0.093	0.773
	tZ9G	-0.098	0.762
	tZOG	-0.098	0.763
	tZR	-0.056	0.863
	tZROG	-0.232	0.467
**Coniferin**	cZ	0.700	0.011
	cZ9G	0.684	0.020
	cZOG	0.723	0.008
	cZR	-0.450	0.142
	cZROG	-0.456	0.136
	DZ	0.404	0.193
	DZ9G	0.436	0.156
	DZOG	0.646	0.023
	DZR	-0.006	0.986
	DZROG	0.624	0.030
	iP	-0.339	0.281
	iP7G	-0.021	0.950
	iP9G	0.615	0.270
	iPR	0.334	0.288
	tZ	-0.087	0.788
	tZ7G	0.704	0.011
	tZ9G	0.704	0.011
	tZOG	0.704	0.011
	tZR	-0.450	0.142
	tZROG	-0.526	0.079
**Dopamine**	cZ	0.460	0.133
	cZ9G	0.438	0.178
	cZOG	0.592	0.043

	cZR	-0.201	0.530
	cZROG	-0.146	0.650
	DZ	0.055	0.866
	DZ9G	0.676	0.016
	DZOG	0.313	0.322
	DZR	-0.111	0.731
	DZROG	0.323	0.306
	iP	-0.082	0.800
	iP7G	-0.617	0.043
	iP9G	0.352	0.562
	iPR	0.203	0.526
	tZ	-0.696	0.012
	tZ7G	0.475	0.119
	tZ9G	0.473	0.120
	tZOG	0.467	0.126
	tZR	-0.202	0.530
	tZROG	-0.512	0.088
**Hypoxanthine**	cZ	0.873	0.000
	cZ9G	0.874	0.000
	cZOG	0.867	0.000
	cZR	-0.384	0.218
	cZROG	-0.450	0.143
	DZ	0.454	0.138
	DZ9G	0.218	0.496
	DZOG	0.867	0.000
	DZR	0.233	0.466
	DZROG	0.818	0.001
	iP	-0.226	0.481
	iP7G	0.075	0.826
	iP9G	0.826	0.085
	iPR	0.544	0.067
	tZ	-0.023	0.943
	tZ7G	0.877	0.000
	tZ9G	0.874	0.000
	tZOG	0.875	0.000
	tZR	-0.384	0.218
	tZROG	-0.549	0.065
**Pipecolinic acid**	cZ	-0.097	0.764
	cZ9G	-0.235	0.486
	cZOG	-0.008	0.980
	cZR	-0.259	0.416
	cZROG	-0.271	0.394
	DZ	-0.238	0.457
	DZ9G	0.807	0.002
	DZOG	-0.327	0.299
	DZR	-0.404	0.192

	DZROG	-0.299	0.345
	iP	-0.393	0.206
	iP7G	-0.497	0.120
	iP9G	-0.538	0.350
	iPR	-0.053	0.871
	tZ	-0.552	0.063
	tZ7G	-0.137	0.670
	tZ9G	-0.144	0.656
	tZOG	-0.148	0.645
	tZR	-0.259	0.416
	tZROG	-0.424	0.169
**Trigonelline**	cZ	-0.236	0.460
	cZ9G	-0.175	0.607
	cZOG	-0.141	0.663
	cZR	0.259	0.416
	cZROG	0.362	0.248
	DZ	-0.325	0.303
	DZ9G	0.379	0.224
	DZOG	-0.342	0.276
	DZR	-0.135	0.676
	DZROG	-0.251	0.432
	iP	0.219	0.493
	iP7G	-0.556	0.076
	iP9G	-0.306	0.617
	iPR	-0.170	0.597
	tZ	-0.488	0.108
	tZ7G	-0.211	0.511
	tZ9G	-0.207	0.518
	tZOG	-0.212	0.508
	tZR	0.259	0.416
	tZROG	0.147	0.648
**Tropine**	cZ	-0.144	0.655
	cZ9G	0.107	0.754
	cZOG	-0.080	0.805
	cZR	0.078	0.811
	cZROG	0.225	0.481
	DZ	-0.249	0.435
	DZ9G	-0.152	0.636
	DZOG	-0.046	0.888
	DZR	-0.064	0.843
	DZROG	0.067	0.836
	iP	0.009	0.979
	iP7G	-0.542	0.085
	iP9G	-0.003	0.996
	iPR	-0.227	0.477
	tZ	-0.226	0.479

	tZ7G	-0.061	0.851
	tZ9G	-0.056	0.862
	tZOG	-0.062	0.848
	tZR	0.078	0.810
	tZROG	0.206	0.520

Table S8. Pearson's correlation analysis of endogenous cytokinins with selected flavonoids.

Flavonoids	Cytokinins	Correlation value	p-value
(-)-Epigallocatechin	cZ	-0.195	0.543
	cZ9G	-0.193	0.569
	cZOG	-0.134	0.678
	cZR	0.435	0.157
	cZROG	0.490	0.106
	DZ	-0.567	0.055
	DZ9G	0.294	0.353
	DZOG	-0.364	0.245
	DZR	0.004	0.990
	DZROG	-0.294	0.354
	iP	0.382	0.221
	iP7G	-0.615	0.044
	iP9G	-0.308	0.615
	iPR	-0.035	0.913
	tZ	-0.517	0.085
	tZ7G	-0.205	0.522
	tZ9G	-0.202	0.529
	tZOG	-0.204	0.525
	tZR	0.435	0.157
	tZROG	0.211	0.511
4-Coumaroylshikimate	cZ	0.065	0.841
	cZ9G	0.068	0.842
	cZOG	-0.039	0.905
	cZR	0.119	0.714
	cZROG	0.108	0.739
	DZ	0.676	0.016
	DZ9G	-0.559	0.059
	DZOG	0.101	0.755
	DZR	0.262	0.411
	DZROG	0.169	0.599
	iP	-0.006	0.986
	iP7G	0.887	0.000
	iP9G	0.953	0.012
	iPR	0.104	0.749
	tZ	0.883	0.000
	tZ7G	0.056	0.862
	tZ9G	0.064	0.843
	tZOG	0.059	0.856
	tZR	0.119	0.714
	tZROG	0.443	0.149
Apiforol	cZ	0.314	0.320
	cZ9G	0.301	0.368
	cZOG	0.406	0.190

	cZR	-0.282	0.374
	cZROG	-0.152	0.636
	DZ	0.416	0.178
	DZ9G	0.229	0.474
	DZOG	0.150	0.641
	DZR	-0.227	0.479
	DZROG	0.253	0.428
	iP	-0.472	0.122
	iP7G	-0.243	0.471
	iP9G	0.513	0.377
	iPR	0.073	0.821
	tZ	-0.349	0.266
	tZ7G	0.318	0.314
	tZ9G	0.324	0.305
	tZOG	0.308	0.330
	tZR	-0.282	0.374
	tZROG	-0.273	0.390
**Chalconaringenin**	cZ	0.796	0.002
	cZ9G	0.782	0.004
	cZOG	0.831	0.001
	cZR	-0.225	0.483
	cZROG	-0.232	0.468
	DZ	0.384	0.218
	DZ9G	0.321	0.309
	DZOG	0.579	0.049
	DZR	0.132	0.683
	DZROG	0.626	0.030
	iP	-0.221	0.490
	iP7G	-0.141	0.680
	iP9G	0.908	0.033
	iPR	0.531	0.075
	tZ	-0.188	0.558
	tZ7G	0.768	0.004
	tZ9G	0.770	0.003
	tZOG	0.763	0.004
	tZR	-0.225	0.482
	tZROG	-0.474	0.119
**Cyanidin**	cZ	-0.695	0.012
	cZ9G	-0.679	0.022
	cZOG	-0.651	0.022
	cZR	0.136	0.674
	cZROG	0.243	0.446
	DZ	0.136	0.674
	DZ9G	-0.403	0.194
	DZOG	-0.728	0.007
	DZR	-0.311	0.325

	DZROG	-0.597	0.040
	iP	0.003	0.992
	iP7G	0.076	0.824
	iP9G	-0.581	0.304
	iPR	-0.405	0.191
	tZ	0.076	0.814
	tZ7G	-0.690	0.013
	tZ9G	-0.687	0.014
	tZOG	-0.691	0.013
	tZR	0.136	0.674
	tZROG	0.263	0.410
	cZ	-0.368	0.239
	cZ9G	-0.340	0.307
	cZOG	-0.353	0.260
	cZR	0.526	0.079
	cZROG	0.549	0.065
	DZ	-0.734	0.007
	DZ9G	0.117	0.717
	DZOG	-0.433	0.160
	DZR	0.062	0.849
**Cyanidin 3-O-(6-O-p-coumaroyl)glucoside**	DZROG	-0.412	0.183
	iP	0.542	0.069
	iP7G	-0.451	0.164
	iP9G	-0.556	0.331
	iPR	-0.169	0.600
	tZ	-0.254	0.426
	tZ7G	-0.372	0.234
	tZ9G	-0.369	0.237
	tZOG	-0.366	0.242
	tZR	0.526	0.079
	tZROG	0.398	0.200
	cZ	0.503	0.096
	cZ9G	0.434	0.183
	cZOG	0.590	0.044
	cZR	-0.257	0.420
	cZROG	-0.206	0.521
	DZ	0.321	0.309
	DZ9G	0.402	0.195
**Cyanidin 3-O-beta-D-glucoside**	DZOG	0.256	0.421
	DZR	-0.095	0.770
	DZROG	0.339	0.281
	iP	-0.368	0.239
	iP7G	-0.432	0.184
	iP9G	0.664	0.221
	iPR	0.364	0.245
	tZ	-0.481	0.114

	tZ7G	0.473	0.120
	tZ9G	0.477	0.117
	tZOG	0.463	0.129
	tZR	-0.257	0.420
	tZROG	-0.411	0.184
**Cyanidin 3-O-beta-D-sambubioside**	cZ	-0.550	0.064
	cZ9G	-0.509	0.110
	cZOG	-0.499	0.099
	cZR	0.160	0.620
	cZROG	0.281	0.377
	DZ	0.188	0.558
	DZ9G	-0.283	0.373
	DZOG	-0.640	0.025
	DZR	-0.219	0.495
	DZROG	-0.437	0.156
	iP	-0.036	0.911
	iP7G	0.023	0.946
	iP9G	-0.328	0.590
	iPR	-0.269	0.398
	tZ	0.156	0.628
	tZ7G	-0.538	0.071
	tZ9G	-0.532	0.075
	tZOG	-0.543	0.068
	tZR	0.160	0.619
	tZROG	0.391	0.208
**Cyanidin 3-O-glucoside**	cZ	-0.429	0.250
	cZ9G	-0.410	0.274
	cZOG	-0.248	0.520
	cZR	-0.331	0.384
	cZROG	-0.192	0.621
	DZ	-0.221	0.567
	DZ9G	0.653	0.056
	DZOG	-0.516	0.155
	DZR	-0.646	0.060
	DZROG	-0.470	0.202
	iP	-0.280	0.466
	iP7G	-0.829	0.006
	iP9G	-0.902	0.098
	iPR	-0.414	0.267
	tZ	-0.850	0.004
	tZ7G	-0.400	0.287
	tZ9G	-0.401	0.285
	tZOG	-0.409	0.275
	tZR	-0.331	0.384
	tZROG	-0.593	0.092
**Delphinidin**	cZ	-0.594	0.042

	cZ9G	-0.529	0.094
	cZOG	-0.591	0.043
	cZR	0.095	0.768
	cZROG	0.179	0.577
	DZ	0.142	0.659
	DZ9G	-0.682	0.015
	DZOG	-0.553	0.062
	DZR	-0.165	0.608
	DZROG	-0.390	0.210
	iP	-0.132	0.683
	iP7G	0.185	0.586
	iP9G	-0.122	0.845
	iPR	-0.295	0.352
	tZ	0.245	0.442
	tZ7G	-0.571	0.052
	tZ9G	-0.570	0.053
	tZOG	-0.574	0.051
	tZR	0.095	0.768
	tZROG	0.302	0.340
**Delphinidin 3-O-beta-D-sambubioside**	cZ	-0.345	0.272
	cZ9G	-0.331	0.320
	cZOG	-0.380	0.223
	cZR	-0.281	0.377
	cZROG	-0.280	0.378
	DZ	0.369	0.238
	DZ9G	-0.371	0.235
	DZOG	-0.198	0.537
	DZR	-0.250	0.433
	DZROG	-0.236	0.461
	iP	-0.324	0.304
	iP7G	0.574	0.065
	iP9G	-0.353	0.560
	iPR	-0.352	0.262
	tZ	0.459	0.133
	tZ7G	-0.337	0.283
	tZ9G	-0.341	0.278
	tZOG	-0.339	0.281
	tZR	-0.281	0.377
	tZROG	-0.025	0.939
**Delphinidin 3-O-glucoside**	cZ	0.713	0.009
	cZ9G	0.685	0.020
	cZOG	0.738	0.006
	cZR	-0.532	0.075
	cZROG	-0.586	0.045
	DZ	0.457	0.135
	DZ9G	0.273	0.390

	DZOG	0.613	0.034
	DZR	0.002	0.995
	DZROG	0.609	0.036
	iP	-0.432	0.161
	iP7G	-0.186	0.585
	iP9G	0.657	0.229
	iPR	0.551	0.064
	tZ	-0.275	0.386
	tZ7G	0.688	0.013
	tZ9G	0.684	0.014
	tZOG	0.685	0.014
	tZR	-0.532	0.075
	tZROG	-0.736	0.006
**Genistein**	cZ	-0.106	0.743
	cZ9G	0.004	0.990
	cZOG	-0.142	0.660
	cZR	0.459	0.134
	cZROG	0.414	0.181
	DZ	-0.627	0.029
	DZ9G	-0.018	0.956
	DZOG	-0.007	0.983
	DZR	0.351	0.263
	DZROG	-0.009	0.979
	iP	0.491	0.105
	iP7G	-0.445	0.170
	iP9G	-0.344	0.571
	iPR	0.148	0.646
	tZ	-0.280	0.379
	tZ7G	-0.081	0.803
	tZ9G	-0.080	0.804
	tZOG	-0.072	0.825
	tZR	0.459	0.134
	tZROG	0.367	0.240
**Homoeriodictyol chalcone**	cZ	0.774	0.003
	cZ9G	0.760	0.007
	cZOG	0.863	0.000
	cZR	-0.355	0.257
	cZROG	-0.371	0.235
	DZ	0.363	0.247
	DZ9G	0.546	0.066
	DZOG	0.647	0.023
	DZR	0.054	0.867
	DZROG	0.632	0.027
	iP	-0.181	0.574
	iP7G	-0.370	0.263
	iP9G	0.774	0.124

	iPR	0.486	0.109
	tZ	-0.471	0.122
	tZ7G	0.775	0.003
	tZ9G	0.771	0.003
	tZOG	0.767	0.004
	tZR	-0.355	0.257
	tZROG	-0.677	0.016
**Kaempferin**	cZ	0.144	0.655
	cZ9G	0.172	0.613
	cZOG	0.277	0.383
	cZR	0.048	0.883
	cZROG	0.180	0.576
	DZ	0.034	0.917
	DZ9G	0.236	0.461
	DZOG	-0.019	0.954
	DZR	-0.109	0.737
	DZROG	0.097	0.764
	iP	-0.026	0.935
	iP7G	-0.636	0.036
	iP9G	0.382	0.526
	iPR	0.082	0.801
	tZ	-0.668	0.018
	tZ7G	0.164	0.611
	tZ9G	0.168	0.601
	tZOG	0.155	0.630
	tZR	0.048	0.883
	tZROG	-0.141	0.661
**Kaempferol 3-O-glucoside**	cZ	0.588	0.044
	cZ9G	0.560	0.073
	cZOG	0.655	0.021
	cZR	-0.321	0.309
	cZROG	-0.280	0.378
	DZ	0.311	0.326
	DZ9G	0.391	0.208
	DZOG	0.419	0.175
	DZR	-0.035	0.914
	DZROG	0.500	0.098
	iP	-0.425	0.169
	iP7G	-0.393	0.232
	iP9G	0.670	0.216
	iPR	0.407	0.189
	tZ	-0.457	0.135
	tZ7G	0.582	0.047
	tZ9G	0.584	0.046
	tZOG	0.575	0.051
	tZR	-0.321	0.309

	tZROG	-0.449	0.143
Leucocyanidin	cZ	0.872	0.000
	cZ9G	0.881	0.000
	cZOG	0.879	0.000
	cZR	-0.393	0.206
	cZROG	-0.470	0.123
	DZ	0.388	0.213
	DZ9G	0.397	0.201
	DZOG	0.762	0.004
	DZR	0.159	0.622
	DZROG	0.716	0.009
	iP	-0.313	0.322
	iP7G	-0.056	0.870
	iP9G	0.810	0.097
	iPR	0.585	0.046
	tZ	-0.197	0.540
	tZ7G	0.846	0.001
	tZ9G	0.842	0.001
	tZOG	0.841	0.001
	tZR	-0.393	0.206
	tZROG	-0.633	0.027
Naringenin	cZ	0.797	0.002
	cZ9G	0.782	0.004
	cZOG	0.829	0.001
	cZR	-0.211	0.510
	cZROG	-0.222	0.488
	DZ	0.367	0.240
	DZ9G	0.318	0.314
	DZOG	0.581	0.048
	DZR	0.146	0.650
	DZROG	0.627	0.029
	iP	-0.206	0.521
	iP7G	-0.141	0.678
	iP9G	0.908	0.033
	iPR	0.541	0.069
	tZ	-0.182	0.572
	tZ7G	0.769	0.003
	tZ9G	0.770	0.003
	tZOG	0.764	0.004
	tZR	-0.211	0.510
	tZROG	-0.461	0.132
Pelargonidin 3,5-di-beta-D-glucoside	cZ	-0.193	0.548
	cZ9G	-0.141	0.680
	cZOG	-0.068	0.833
	cZR	0.239	0.455
	cZROG	0.393	0.206

	DZ	-0.436	0.156
	DZ9G	0.205	0.523
	DZOG	-0.335	0.287
	DZR	-0.259	0.416
	DZROG	-0.298	0.347
	iP	0.216	0.501
	iP7G	-0.544	0.084
	iP9G	-0.195	0.753
	iPR	-0.396	0.202
	tZ	-0.503	0.096
	tZ7G	-0.172	0.592
	tZ9G	-0.170	0.596
	tZOG	-0.177	0.582
	tZR	0.239	0.455
	tZROG	-0.010	0.976
**Proanthocyanidin B2**	cZ	-0.323	0.306
	cZ9G	-0.256	0.447
	cZOG	-0.226	0.481
	cZR	-0.050	0.878
	cZROG	0.015	0.963
	DZ	-0.193	0.547
	DZ9G	-0.019	0.954
	DZOG	-0.376	0.228
	DZR	-0.231	0.469
	DZROG	-0.209	0.515
	iP	-0.115	0.721
	iP7G	-0.640	0.034
	iP9G	-0.250	0.685
	iPR	-0.026	0.937
	tZ	-0.557	0.060
	tZ7G	-0.290	0.361
	tZ9G	-0.295	0.351
	tZOG	-0.293	0.355
	tZR	-0.050	0.878
	tZROG	-0.221	0.489

Table S9. Pearson's correlation analysis of endogenous cytokinins with selected phenolic compounds.

Phenolic Compounds	Cytokinins	Correlation Value	p-value
**1-O-Sinapoyl-beta-D-glucose**	cZ	0.176	0.584
	cZ9G	0.223	0.509
	cZOG	0.280	0.379
	cZR	-0.020	0.951
	cZROG	0.036	0.911
	DZ	-0.017	0.957
	DZ9G	0.352	0.262
	DZOG	0.061	0.849
	DZR	-0.012	0.971
	DZROG	0.188	0.558
	iP	-0.147	0.648
	iP7G	-0.642	0.033
	iP9G	0.285	0.643
	iPR	0.249	0.435
	tZ	-0.697	0.012
	tZ7G	0.202	0.529
	tZ9G	0.201	0.530
	tZOG	0.192	0.549
	tZR	-0.020	0.951
	tZROG	-0.204	0.525
**3-O-Methylgallate**	cZ	0.369	0.238
	cZ9G	0.338	0.309
	cZOG	0.427	0.166
	cZR	0.110	0.734
	cZROG	0.097	0.765
	DZ	-0.144	0.655
	DZ9G	0.269	0.398
	DZOG	0.316	0.317
	DZR	0.143	0.657
	DZROG	0.216	0.499
	iP	0.457	0.135
	iP7G	-0.165	0.629
	iP9G	0.227	0.713
	iPR	0.075	0.817
	tZ	-0.090	0.781
	tZ7G	0.374	0.231
	tZ9G	0.372	0.234
	tZOG	0.371	0.235

	tZR	0.110	0.735
	tZROG	-0.195	0.544
**5-O-Caffeoylshikimic acid**	cZ	0.138	0.668
	cZ9G	0.063	0.853
	cZOG	0.305	0.334
	cZR	-0.174	0.589
	cZROG	-0.110	0.733
	DZ	-0.378	0.226
	DZ9G	0.561	0.058
	DZOG	-0.023	0.944
	DZR	-0.329	0.296
	DZROG	-0.086	0.789
	iP	0.005	0.989
	iP7G	-0.927	0.000
	iP9G	-0.312	0.610
	iPR	-0.071	0.825
	tZ	-0.944	0.000
	tZ7G	0.130	0.686
	tZ9G	0.125	0.700
	tZOG	0.120	0.710
	tZR	-0.174	0.589
	tZROG	-0.614	0.034
**Caffeic acid**	cZ	-0.366	0.242
	cZ9G	-0.364	0.271
	cZOG	-0.357	0.255
	cZR	-0.220	0.493
	cZROG	-0.168	0.601
	DZ	0.125	0.698
	DZ9G	0.069	0.832
	DZOG	-0.406	0.190
	DZR	-0.341	0.278
	DZROG	-0.305	0.335
	iP	-0.589	0.044
	iP7G	-0.059	0.864
	iP9G	-0.369	0.541
	iPR	-0.165	0.609
	tZ	-0.175	0.585
	tZ7G	-0.377	0.227
	tZ9G	-0.375	0.229
	tZOG	-0.381	0.221
	tZR	-0.219	0.493
	tZROG	0.030	0.926
**Cinnamaldehyde**	cZ	-0.077	0.812

	cZ9G	-0.330	0.321
	cZOG	-0.101	0.754
	cZR	-0.420	0.174
	cZROG	-0.487	0.108
	DZ	0.063	0.845
	DZ9G	0.487	0.108
	DZOG	-0.231	0.470
	DZR	-0.362	0.248
	DZROG	-0.257	0.419
	iP	-0.675	0.016
	iP7G	-0.066	0.847
	iP9G	-0.488	0.404
	iPR	0.077	0.811
	tZ	-0.260	0.414
	tZ7G	-0.162	0.616
	tZ9G	-0.164	0.611
	tZOG	-0.164	0.611
	tZR	-0.420	0.174
	tZROG	-0.328	0.298
**Coniferin**	cZ	-0.243	0.446
	cZ9G	-0.243	0.471
	cZOG	-0.118	0.715
	cZR	-0.058	0.858
	cZROG	0.026	0.936
	DZ	-0.066	0.838
	DZ9G	0.345	0.272
	DZOG	-0.392	0.207
	DZR	-0.294	0.353
	DZROG	-0.269	0.398
	iP	-0.223	0.485
	iP7G	-0.605	0.049
	iP9G	-0.266	0.665
	iPR	-0.060	0.853
	tZ	-0.632	0.027
	tZ7G	-0.239	0.454
	tZ9G	-0.239	0.454
	tZOG	-0.253	0.428
	tZR	-0.058	0.858
	tZROG	-0.104	0.748
**Coniferyl alcohol**	cZ	-0.089	0.784
	cZ9G	-0.022	0.948
	cZOG	0.016	0.960
	cZR	-0.066	0.840

	cZROG	-0.013	0.968
	DZ	-0.208	0.517
	DZ9G	0.390	0.210
	DZOG	-0.161	0.617
	DZR	-0.169	0.599
	DZROG	-0.050	0.876
	iP	-0.201	0.531
	iP7G	-0.683	0.020
	iP9G	-0.273	0.657
	iPR	0.041	0.900
	tZ	-0.721	0.008
	tZ7G	-0.057	0.860
	tZ9G	-0.061	0.850
	tZOG	-0.066	0.840
	tZR	-0.066	0.840
	tZROG	-0.228	0.477
**Coniferyl aldehyde**	cZ	0.102	0.752
	cZ9G	-0.229	0.498
	cZOG	0.082	0.800
	cZR	-0.492	0.104
	cZROG	-0.582	0.047
	DZ	0.174	0.589
	DZ9G	0.551	0.063
	DZOG	-0.087	0.788
	DZR	-0.333	0.291
	DZROG	-0.144	0.656
	iP	-0.673	0.016
	iP7G	-0.048	0.890
	iP9G	-0.355	0.558
	iPR	0.197	0.539
	tZ	-0.267	0.401
	tZ7G	0.002	0.995
	tZ9G	-0.001	0.998
	tZOG	-0.001	0.997
	tZR	-0.492	0.104
	tZROG	-0.469	0.124
**Ferulic acid**	cZ	-0.343	0.275
	cZ9G	-0.347	0.296
	cZOG	-0.305	0.335
	cZR	-0.215	0.502
	cZROG	-0.188	0.559
	DZ	-0.042	0.898
	DZ9G	0.297	0.349

	DZOG	-0.394	0.205
	DZR	-0.339	0.281
	DZROG	-0.316	0.317
	iP	-0.502	0.096
	iP7G	-0.269	0.424
	iP9G	-0.474	0.420
	iPR	-0.129	0.690
	tZ	-0.370	0.236
	tZ7G	-0.350	0.265
	tZ9G	-0.352	0.262
	tZOG	-0.357	0.255
	tZR	-0.215	0.502
	tZROG	-0.072	0.825
**Gentisic acid**	cZ	-0.389	0.212
	cZ9G	-0.316	0.344
	cZOG	-0.346	0.271
	cZR	-0.268	0.400
	cZROG	-0.207	0.518
	DZ	-0.027	0.933
	DZ9G	0.169	0.599
	DZOG	-0.235	0.461
	DZR	-0.311	0.325
	DZROG	-0.241	0.450
	iP	-0.265	0.405
	iP7G	-0.284	0.397
	iP9G	-0.395	0.511
	iPR	-0.276	0.384
	tZ	-0.186	0.562
	tZ7G	-0.354	0.260
	tZ9G	-0.350	0.264
	tZOG	-0.356	0.255
	tZR	-0.268	0.401
	tZROG	0.131	0.685
**L-Phenylalanine**	cZ	-0.356	0.256
	cZ9G	-0.370	0.263
	cZOG	-0.426	0.168
	cZR	-0.189	0.556
	cZROG	-0.192	0.551
	DZ	0.384	0.218
	DZ9G	-0.155	0.630
	DZOG	-0.324	0.305
	DZR	-0.197	0.540
	DZROG	-0.261	0.413

	iP	-0.544	0.067
	iP7G	0.504	0.114
	iP9G	-0.337	0.579
	iPR	-0.158	0.624
	tZ	0.368	0.240
	tZ7G	-0.377	0.227
	tZ9G	-0.374	0.231
	tZOG	-0.379	0.225
	tZR	-0.189	0.556
	tZROG	0.228	0.476
**Methylthioadenosine**	cZ	0.016	0.960
	cZ9G	-0.162	0.635
	cZOG	-0.139	0.668
	cZR	-0.241	0.450
	cZROG	-0.364	0.245
	DZ	0.413	0.183
	DZ9G	-0.132	0.683
	DZOG	0.052	0.873
	DZR	0.041	0.898
	DZROG	-0.022	0.946
	iP	-0.378	0.225
	iP7G	0.727	0.011
	iP9G	-0.051	0.935
	iPR	0.193	0.548
	tZ	0.586	0.045
	tZ7G	-0.062	0.848
	tZ9G	-0.057	0.860
	tZOG	-0.054	0.867
	tZR	-0.241	0.450
	tZROG	0.168	0.601
**p-Coumaroyl quinic acid**	cZ	-0.182	0.572
	cZ9G	-0.255	0.449
	cZOG	-0.302	0.341
	cZR	-0.122	0.707
	cZROG	-0.153	0.635
	DZ	0.491	0.105
	DZ9G	-0.313	0.322
	DZOG	-0.145	0.652
	DZR	-0.073	0.822
	DZROG	-0.166	0.607
	iP	-0.380	0.223
	iP7G	0.878	0.000
	iP9G	-0.351	0.562

	tZ	0.710	0.010
	tZ7G	-0.225	0.481
	tZ9G	-0.221	0.490
	tZOG	-0.222	0.487
	tZR	-0.122	0.707
	tZROG	0.270	0.396
**Sinapaldehyde**	cZ	0.000	1.000
	cZ9G	-0.166	0.625
	cZOG	-0.003	0.993
	cZR	-0.428	0.165
	cZROG	-0.490	0.106
	DZ	0.015	0.964
	DZ9G	0.520	0.083
	DZOG	-0.115	0.722
	DZR	-0.307	0.332
	DZROG	-0.128	0.692
	iP	-0.636	0.026
	iP7G	-0.237	0.483
	iP9G	-0.373	0.536
	iPR	0.139	0.666
	tZ	-0.393	0.206
	tZ7G	-0.059	0.855
	tZ9G	-0.063	0.846
	tZOG	-0.062	0.847
	tZR	-0.428	0.165
	tZROG	-0.388	0.212
**Sinapyl alcohol**	cZ	-0.262	0.411
	cZ9G	-0.234	0.489
	cZOG	-0.351	0.264
	cZR	-0.174	0.590
	cZROG	-0.141	0.662
	DZ	0.454	0.138
	DZ9G	-0.449	0.143
	DZOG	-0.159	0.621
	DZR	-0.147	0.648
	DZROG	-0.141	0.662
	iP	-0.492	0.104
	iP7G	0.725	0.012
	iP9G	-0.127	0.838
	iPR	-0.280	0.378
	tZ	0.600	0.039
	tZ7G	-0.268	0.399
	tZ9G	-0.263	0.409

	tZOG	-0.268	0.399
	tZR	-0.174	0.590
	tZROG	0.291	0.359
Tetrahydrofolate	cZ	-0.411	0.238
	cZ9G	-0.579	0.102
	cZOG	-0.315	0.376
	cZR	-0.002	0.996
	cZROG	0.037	0.919
	DZ	-0.230	0.523
	DZ9G	0.211	0.559
	DZOG	-0.585	0.076
	DZR	-0.305	0.391
	DZROG	-0.549	0.100
	iP	-0.217	0.548
	iP7G	-0.629	0.069
	iP9G	-0.630	0.566
	iPR	0.101	0.781
	tZ	-0.684	0.029
	tZ7G	-0.476	0.164
	tZ9G	-0.486	0.155
	tZOG	-0.477	0.163
	tZR	-0.002	0.996
	tZROG	-0.193	0.593
trans-5-O-(4-Coumaroyl)-D-quinate	cZ	-0.058	0.859
	cZ9G	-0.092	0.789
	cZOG	-0.178	0.579
	cZR	-0.170	0.598
	cZROG	-0.189	0.557
	DZ	0.407	0.189
	DZ9G	-0.386	0.215
	DZOG	-0.001	0.998
	DZR	-0.044	0.892
	DZROG	-0.022	0.946
	iP	-0.479	0.115
	iP7G	0.779	0.005
	iP9G	0.137	0.826
	tZ	0.630	0.028
	tZ7G	-0.090	0.781
	tZ9G	-0.086	0.790
	tZOG	-0.088	0.785
	tZR	-0.170	0.598
	tZROG	0.194	0.547
Vanillic acid	cZ	0.205	0.522

	cZ9G	0.243	0.471
	cZOG	0.222	0.488
	cZR	-0.192	0.551
	cZROG	-0.198	0.538
	DZ	0.622	0.031
	DZ9G	0.077	0.812
	DZOG	0.212	0.508
	DZR	0.081	0.802
	DZROG	0.318	0.313
	iP	-0.270	0.397
	iP7G	0.255	0.448
	iP9G	0.367	0.544
	iPR	0.242	0.449
	tZ	0.162	0.616
	tZ7G	0.234	0.464
	tZ9G	0.234	0.464
	tZOG	0.226	0.479
	tZR	-0.192	0.551
	tZROG	-0.073	0.822

Table S10. Pathway analysis, encompassing all metabolic pathways from KEGG and HMDB databases with total hits for *D. dentatus'* red and white ecotypes. Pathways are categorized based on their p-values (on the y-axis), derived from pathway enrichment analysis, and pathway impact values (on the x-axis), obtained from pathway topology analysis. The analysis was performed using the MetaboAnalyst 6.0 platform with the GSEA algorithm, using default settings, and the *Arabidopsis thaliana* pathway library.

Pathways	Total	Expected	Hits	P value	Impact
Monobactam biosynthesis	8	1.7493	3	0.24515	1
Linoleic acid metabolism	4	0.87467	3	0.034762	1
Isoquinoline alkaloid biosynthesis	6	1.312	4	0.023146	1
Flavone and flavonol biosynthesis	10	2.1867	7	0.001463	1
Arachidonic acid metabolism	9	1.968	5	0.028017	1
Tyrosine metabolism	17	3.7173	13	2.15E-06	0.89945
Alanine, aspartate and glutamate metabolism	22	4.8107	7	0.1867	0.79136
One carbon pool by folate	8	1.7493	5	0.015126	0.76191
Anthocyanin biosynthesis	11	2.4053	6	0.017721	0.66666
Phenylalanine metabolism	12	2.624	4	0.25659	0.61539
Fructose and mannose metabolism	18	3.936	4	0.57807	0.53233
alpha-Linolenic acid metabolism	26	5.6853	10	0.039767	0.5061
C5-Branched dibasic acid metabolism	6	1.312	3	0.12272	0.5
Amino sugar and nucleotide sugar metabolism	52	11.371	14	0.22932	0.46996
Arginine and proline metabolism	32	6.9973	9	0.25088	0.4643
Riboflavin metabolism	13	2.8427	4	0.31134	0.45927
Phenylpropanoid biosynthesis	43	9.4027	16	0.014646	0.43304
Starch and sucrose metabolism	22	4.8107	7	0.1867	0.42611
Pantothenate and CoA biosynthesis	25	5.4667	7	0.2958	0.42105
Phenylalanine, tyrosine and tryptophan biosynthesis	22	4.8107	9	0.033666	0.41942
Lysine biosynthesis	9	1.968	3	0.3119	0.41892
Arginine biosynthesis	18	3.936	6	0.18152	0.39904
Diterpenoid biosynthesis	29	6.3413	11	0.035085	0.39731
Valine, leucine and isoleucine biosynthesis	22	4.8107	11	0.003152	0.39451
beta-Alanine metabolism	18	3.936	6	0.18152	0.39286
Ascorbate and aldarate metabolism	20	4.3733	8	0.05078	0.37755
Monoterpenoid biosynthesis	7	1.5307	6	0.000602	0.375
Tryptophan metabolism	29	6.3413	7	0.45492	0.37405
Galactose metabolism	27	5.904	10	0.051308	0.37089
Butanoate metabolism	17	3.7173	7	0.057056	0.36363
Terpenoid backbone biosynthesis	30	6.56	10	0.09839	0.35742
Inositol phosphate metabolism	28	6.1227	9	0.13725	0.34252
Vitamin B6 metabolism	12	2.624	6	0.029044	0.29524
Flavonoid biosynthesis	47	10.277	20	0.00099	0.29269
Ubiquinone and other terpenoid-quinone biosynthesis	47	10.277	8	0.84055	0.29106
Sphingolipid metabolism	27	5.904	5	0.73667	0.28449
Porphyrin metabolism	48	10.496	7	0.92778	0.2802
Glyoxylate and dicarboxylate metabolism	29	6.3413	7	0.45492	0.27426
Pentose phosphate pathway	19	4.1547	6	0.21947	0.26972
Citrate cycle (TCA cycle)	20	4.3733	6	0.25977	0.26883
Pyruvate metabolism	23	5.0293	2	0.97528	0.26542
Glycolysis / Gluconeogenesis	26	5.6853	5	0.70365	0.26266

Cysteine and methionine metabolism	47	10.277	8	0.84055	0.25018
Selenocompound metabolism	13	2.8427	3	0.56604	0.2437
Cyanoamino acid metabolism	29	6.3413	9	0.16276	0.23729
Purine metabolism	73	15.963	12	0.90594	0.23307
Sesquiterpenoid and triterpenoid biosynthesis	24	5.248	9	0.058619	0.2183
Pentose and glucuronate interconversions	17	3.7173	5	0.30727	0.21429
Glycosylphosphatidylinositol (GPI)-anchor biosynthesis	14	3.0613	1	0.96893	0.19005
Lysine degradation	20	4.3733	4	0.66742	0.16667
Propanoate metabolism	19	4.1547	4	0.62442	0.14357
Histidine metabolism	16	3.4987	5	0.25999	0.14286
Glucosinolate biosynthesis	65	14.213	13	0.69346	0.13784
Glycine, serine and threonine metabolism	33	7.216	8	0.43691	0.13205
Valine, leucine and isoleucine degradation	37	8.0907	10	0.27711	0.12512
Glycerolipid metabolism	21	4.592	2	0.96231	0.11766
Glycerophospholipid metabolism	38	8.3093	3	0.99478	0.11559
Glutathione metabolism	26	5.6853	9	0.093096	0.10211
Pyrimidine metabolism	41	8.9653	8	0.70417	0.08859
Biosynthesis of various plant secondary metabolites	29	6.3413	3	0.96925	0.08
Sulfur metabolism	15	3.28	1	0.97579	0.07459
Folate biosynthesis	31	6.7787	3	0.97901	0.05303
Carbon fixation in photosynthetic organisms	21	4.592	4	0.70693	0.05025
Fatty acid degradation	37	8.0907	3	0.9936	0.03122
Zeatin biosynthesis	21	4.592	7	0.15466	0.02034
Nicotinate and nicotinamide metabolism	13	2.8427	1	0.96014	0.0202
Lipoic acid metabolism	24	5.248	5	0.62939	0.01939
Carotenoid biosynthesis	43	9.4027	4	0.99196	0.01264
Fatty acid biosynthesis	56	12.245	7	0.97689	0.01123
N-Glycan biosynthesis	35	7.6533	2	0.99824	0.00978
Thiamine metabolism	22	4.8107	4	0.74293	0.00578
Tropane, piperidine and pyridine alkaloid biosynthesis	9	1.968	4	0.11166	0
Steroid biosynthesis	44	9.6213	2	0.99978	0
Phosphonate and phosphinate metabolism	5	1.0933	1	0.70935	0
Nitrogen metabolism	12	2.624	2	0.77554	0
Indole alkaloid biosynthesis	4	0.87467	1	0.62773	0
Glycosphingolipid biosynthesis - globo and isoglobo series	6	1.312	1	0.77312	0
Fatty acid elongation	23	5.0293	3	0.90825	0
D-Amino acid metabolism	7	1.5307	2	0.47425	0
Cutin, suberine and wax biosynthesis	18	3.936	3	0.78942	0
Brassinosteroid biosynthesis	26	5.6853	1	0.99846	0
Biosynthesis of unsaturated fatty acids	22	4.8107	8	0.086002	0
Betalain biosynthesis	3	0.656	1	0.52328	0

References

1. Persson, B.C.; Björk, G. Isolation of the gene (miaE) encoding the hydroxylase involved in the synthesis of 2-methylthio-cis-ribozeatin in tRNA of Salmonella typhimurium and characterization of mutants. *Journal of bacteriology* **1993**, *175*, 7776-7785.
2. Yonekura-Sakakibara, K.; Kojima, M.; Yamaya, T.; Sakakibara, H. Molecular characterization of cytokinin-responsive histidine kinases in maize. Differential ligand preferences and response to cis-zeatin. *Plant physiology* **2004**, *134*, 1654-1661.
3. Frebort, I.; Kowalska, M.; Hluska, T.; Frébortová, J.; Galuszka, P. Evolution of cytokinin biosynthesis and degradation. *Journal of Experimental Botany* **2011**, *62*, 2431-2452.
4. Pertry, I.; Václavíková, K.; Depuydt, S.; Galuszka, P.; Spíchal, L.; Temmerman, W.; Stes, E.; Schmülling, T.; Kakimoto, T.; Van Montagu, M.C. Identification of Rhodococcus fascians cytokinins and their modus operandi to reshape the plant. *Proceedings of the National Academy of Sciences* **2009**, *106*, 929-934.
5. Morrison, E.N.; Donaldson, M.E.; Saville, B.J. Identification and analysis of genes expressed in the Ustilago maydis dikaryon: uncovering a novel class of pathogenesis genes. *Canadian journal of plant pathology* **2012**, *34*, 417-435.
6. Hošek, P.; Hoyerová, K.; Kiran, N.S.; Dobrev, P.I.; Zahajská, L.; Filepová, R.; Motyka, V.; Müller, K.; Kamínek, M. Distinct metabolism of N-glucosides of isopentenyladenine and trans-zeatin determines cytokinin metabolic spectrum in Arabidopsis. *New Phytologist* **2020**, *225*, 2423-2438.
7. Márquez-López, R.E.; Quintana-Escobar, A.O.; Loyola-Vargas, V.M. Cytokinins, the Cinderella of plant growth regulators. *Phytochemistry Reviews* **2019**, *18*, 1387-1408.
8. Morrison, E.N.; Emery, R.N.; Saville, B.J. Phytohormone involvement in the Ustilago maydis–Zea mays pathosystem: relationships between abscisic acid and cytokinin levels and strain virulence in infected cob tissue. *PLoS One* **2015**, *10*, e0130945.
